# Comparative Analysis of Extracellular Vesicle‐Like Particles From Different Processing *Gastrodia elata* Bl.: Physicochemical Properties, Biosafety, and Neuroprotection Potential as a Functional Food Ingredient

**DOI:** 10.1002/fsn3.72044

**Published:** 2026-06-23

**Authors:** Yuanyuan Qin, Shuming Li, Jia Yu, Jingyu Weng, Bo Li, Xinyi Liu, Ke Wang, Yuangui Yang, Honghong Jiao, Jiaofeng Wu, Hongbo Xu

**Affiliations:** ^1^ Shaanxi Collaborative Innovation Center of Chinese Medicinal Resources Industrialization Shaanxi University of Chinese Medicine Xianyang People's Republic of China; ^2^ School of Medical Engineering Haojing College of Shaanxi University of Science & Technology Xianyang Shaanxi People's Republic of China

**Keywords:** extracellular vesicle‐like particles, food processing, functional food, *Gastrodiae elata*, medicine‐food homology, neuroprotection

## Abstract

*Gastrodia elata* (GE) is a classic medicine‐food homologous plant that has long been utilized in dietary practices for anti‐inflammation and neuroprotection, as well as improving cerebrovascular circulation. In recent years, extracellular vesicle‐like particles (EVLPs) derived from edible plants have emerged as a novel class of dietary bioactive ingredients and natural nanocarriers, demonstrating broad potential in health‐related applications. However, how food processing technologies influence the characteristics of such EVLPs remains unclear. To address this, the present study aims to isolate EVLPs from three processed GE products—dried GE (DGE), wine‐processed GE (WGE), and ginger‐processed GE (GGE)—and systematically evaluate their physicochemical properties, biosafety, and potential functions in neurological health. The morphology, particle size, concentration, and zeta potential of three GE‐EVLPs were characterized. Their bioactive molecular composition was analyzed using untargeted metabolomics. Biosafety was comprehensively evaluated through cytotoxicity assays, hemolysis tests, and histopathological analysis. The potential of these EVLPs in supporting neurological health was further assessed in an oxygen–glucose deprivation/reoxygenation (OGD/R) cell model, with a focus on cell viability and immunomodulatory function. Our results confirm the presence of EVLPs in all three processed GE products and demonstrate that the processing methods significantly influence their physical properties, functional components, and health‐promoting efficacy. Notably, WGE‐EVLPs exhibited the greatest development potential owing to their smaller particle size, superior cellular uptake efficiency, and enhanced protective effects. This study provides novel insights into how food processing modulates the functional properties of edible plant‐derived EVLPs and highlights their promise as functional food ingredients or dietary nanocarriers for neurological health support.

## Graphical abstract

This study systematically characterizes extracellular vesicle‐like particles (EVLPs) from three processed *Gastrodia elata* forms (dry, wine, and ginger), evaluating their physicochemical properties, metabolite profiles, biocompatibility, and neuroprotective efficacy in an OGD/R model, highlighting their neuroprotective potential as a functional food ingredient.

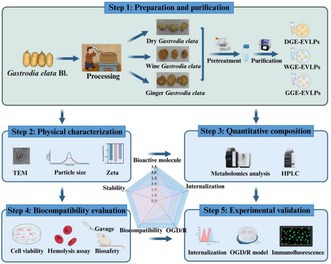

## 1 Introduction

*Gastrodia elata* (GE) Blume, known as a classic representative of “medicine‐food homology” in traditional Chinese medicine (TCM), possesses both significant medicinal value and edible value. Its tubers (the commonly consumed part) are rich in nutrients, have well‐documented health‐promoting properties, and effectively retain bioactive ingredients (Jia et al. 2025). Owing to these characteristics, the application of GE in food processing and the nutraceutical industry has been increasingly widespread (Gong et al. 2024; Li et al. 2025; Su et al. 2024). These tubers contain polysaccharides, gastrodin, parishins, phenolic derivatives, and other bioactive compounds that confer antioxidant, anti‐inflammatory, and neuroprotective activities (Wen et al. 2025). In both traditional practice and modern food processing, GE is often processed to enhance its efficacy, reduce potential side effects, and improve palatability or shelf‐life (Yang et al. 2024), while also facilitating storage to meet the demands of clinical practice in TCM. The processing history of GE can be traced to the Northern and Southern Dynasties. The common processed forms for GE include dried *Gastrodiae elata* (DGE), wine‐processed *Gastrodiae elata* (WGE), and ginger‐processed *Gastrodiae elata* (GGE). These techniques are known to alter the chemical profile of bioactive compounds, suggesting that processing may fundamentally influence the functional properties of GE.

In recent years, edible plant‐derived extracellular vesicle‐like nanoparticles (EVLPs) from edible sources have gained significant attention in the field of functional food science (Xiao et al. 2024; Zhan et al. 2023). These naturally sourced EVLPs possess inherent advantages such as abundant resources and relatively simple preparation (Watabe et al. 2025; Zou et al. 2024). Besides, they are rich in various active components, including proteins, nucleic acids, active small molecules (Huang et al. 2025; Khadilkar et al. 2025), and lipids, exhibiting characteristics of multi‐target synergistic actions (Han et al. 2025; Su et al. 2023). Given their excellent biocompatibility, EVLPs have emerged as a promising strategy to overcome the challenge of low bioavailability that plagues many active ingredients due to their complex molecular structures and poor liposolubility (Huang et al. 2021; Jang et al. 2023). Research has shown that EVLPs derived from *Panax notoginseng* can effectively alleviate ischemia/reperfusion injury following spinal cord injury and promote functional neurological recovery (Li et al. 2023).

However, whether the food processing procedure influences the edible plants‐derived EVLPs was unclear, including the existence, content, and even biological activities (Song et al. 2025). Therefore, this study used GE as a model medicine‐food homologous plant to systematically investigate the effects of different processing methods (drying, wine‐processing, ginger‐processing) on the corresponding EVLPs (DGE‐EVLPs, WGE‐EVLPs, GGE‐EVLPs) (Liu et al. 2025). This work aims to comprehensively compare their basic physicochemical characteristics, active composition, biosafety, and particularly their neuroprotective potential, thereby providing a scientific basis for the high‐value utilization of processed GE in functional foods and dietary supplements.

## 2 Materials and Methods

### 2.1 Materials

#### 2.1.1 Animals and Cells

The murine microglial cell (BV2) line was obtained from the Cell Bank of the Chinese Medicine Innovation Center, Shaanxi University of Chinese Medicine. Male Sprague–Dawley (SD) rats, weighing 200 ± 10 g, were supplied by Chengdu Dashuo Experimental Animal Co. Ltd. (animal production license number: SCXK (Chuan) 2020‐0030). All experimental procedures were approved by the Animal Ethics Committee of Shaanxi University of Chinese Medicine and conducted in accordance with the Management Regulations of Experimental Animals.

#### 2.1.2 Reagents

Fresh rhizomes of GE were procured from Hanzhong, Shaanxi Province, China. DMEM medium (lot number: 6125099) was obtained from Thermo Fisher Scientific (Suzhou) Instrument Co. Ltd. TransSerum FQ Fetal Bovine Serum (lot number: R20228) and Easy II Protein Quantitative Kit (BCA) (lot number: S11119) were purchased from TransGen Biotech Co. Ltd. CCK‐8 assay kit (lot number: 2500030003) was acquired from Solarbio Science & Technology Co. Ltd. BeyoExo Exosome Labeling and Tracking Kit (PKH67) (lot number: A235250515) was supplied by Beyotime Biotechnology. Cell Membrane Red Fluorescent Probe (DiI) (lot number: JS254333), DAPI stain (lot number: J02IS216668), Simulated Gastric Fluid (lot number: KR44635A) and Simulated Intestinal Fluid (lot number: KR44293A) were sourced from Yuanye Bio‐Technology Co. Ltd. CD86 rabbit monoclonal antibody (lot number: BE02176801) was provided by Biosynthesis Biotechnology Co. Ltd. Rapid RNA extraction kit (lot number: A6A4324), Evo M‐MLV Reverse Transcription Kit (lot number: A6A3359) and SYBR Green Pro Taq HS Premixed qPCR Kit (lot number: A6A3118) were purchased from Xi'an Hailing Biological Engineering Co. Ltd. CD206 polyclonal antibody (lot number: 00178078), Goat Anti‐Rabbit IgG Green Fluorescent secondary antibody (lot number: 20001254), and Goat Anti‐Rabbit IgG Red Fluorescent secondary antibody (lot number: 20001397) were all obtained from Wuhan Boster Biological Technology Co. Ltd. MiRNeasy Mini Kit (50) (lot number: 175019462) was purchased from QIAGEN GmbH, Germany. Gastrodin (lot number: M11GB148059), 4‐Hydroxybenzyl alcohol (lot number: R09D8X50333), 4‐Hydroxybenzaldehyde (lot number: Y02J7C15574), Parishin A (lot number: M09HB177711), and Parishin B (lot number: JB258636) were acquired from Shanghai Yuanye Bio‐Technology Co. Ltd. Parishin C (lot number: wkq22061402) was procured from Sichuan Vicki Biotechnology Co. Ltd. Parishin E was bought from Baoji Chenguang Biotechnology Co. Ltd. HPLC‐grade acetonitrile was supplied by Fisher Scientific, USA.

#### 2.1.3 Instruments

Fully automated exosome extraction system (EXODUS H‐600, Shenzhen Huixin Biotech Co. Ltd., China), Exosome isolation and enrichment nanochip (EID Exosome Enrichment Chip; Shenzhen Huixin Biotech Co. Ltd., China), Transmission electron microscope (TECNAI G2 12; FEI Company, USA), Nanoparticle Tracking Analysis (NTA) (ZetaView PMX‐120, Particle Metrix, Germany), Flow cytometer (FACSVerse, BD Biosciences, USA), High‐content analysis system (Operetta CLS; PerkinElmer, UK), CO_2_ incubator (TY2019001798, Thermo Fisher Scientific, USA), inverted microscope (Olympus IX73, Olympus Corporation, Japan), High‐performance liquid chromatography (HPLC) system (LC‐2030C, Shimadzu Corporation, Japan).

### 2.2 Methods

#### 2.2.1 Preparation of DGE‐EVLPs, WGE‐EVLPs, and GGE‐EVLPs

Fresh GE rhizomes were steamed at 100°C for 60 min, immediately sectioned into 3–4 mm slices, and desiccated at 60°C to afford raw‐dried *Gastrodin elata* (DGE). DGE was separately blended with fresh ginger juice (10:1, v/w) and rice wine (5:1, v/w), moistened in a sealed vessel until the adjuvants were fully absorbed, and then stir‐baked over gentle heat until the liquids were completely exhausted, yielding GGE and WGE, respectively (Su et al. 2024). Each processed GE sample was mixed with sterile PBS (0.01 M, pH 7.4) at a sample/PBS ratio of 1:10 (w/v; 10.0 g sample powder in 100 mL PBS). The mixture was vortexed for 5 min and homogenized on ice, followed by sequential centrifugation at 3000×*g* for 10, 6000×*g* for 20, and 10,000×*g* for 30 min at 4°C. The supernatant was sequentially filtered through 0.45 and 0.22 μm sterile PES membranes using low‐vacuum filtration, and subsequently purified using the Exosome Detection and Ultra‐rapid Separation System (Exodus), affording DGE‐EVLPs, WGE‐EVLPs, and GGE‐EVLPs, respectively (Hu et al. 2026; Ma et al. 2026).

#### 2.2.2 Characterization and Identification of the Three EVLPs

##### 2.2.2.1 Observation by Transmission Electron Microscopy (TEM)

The three EVLPs preparations were diluted in PBS to a final protein‐equivalent concentration of 100 μg/mL; 10 μL of each suspension were spotted onto carbon‐coated copper grids and incubated for 10 min to facilitate particle adhesion. After wicking away unbound sample with filter paper, the grids were stained with 2% uranyl acetate for 3 min. Following blotting of excess stain, the specimens were desiccated in air for 20 min prior to TEM examination at an accelerating voltage of 100 kV (Huang et al. 2021).

##### 2.2.2.2 Particle Size and Zeta Potential Analysis

For NTA and zeta‐potential measurement, 10 μL of EVLPs suspension was diluted with 990 μL filtered PBS, and the final concentration was adjusted to the recommended measurable range. Measurements were conducted at 25°C using PBS as the dispersant (Jang et al. 2023). The refractive index was set at 1.333 and the viscosity was set at 0.8872 cP. The particle refractive index was set at 1.45 for all three EVLPs samples, consistent with lipid/protein vesicle‐like nanoparticles. Each sample was measured in triplicate.

#### 2.2.3 Protein Concentration Measurement

For protein measurement, 50 μL of EVLPs suspension was mixed with 150 μL lysis buffer on ice for 30 min and centrifuged at 12,000×*g* (approximately 13,400 rpm with the rotor used in this study) for 10 min at 4°C. Then, 20 μL standards or samples were mixed with 200 μL BCA working solution and incubated at 37°C for 30 min before absorbance detection at 562 nm (Zhao et al. 2023). Sample protein concentrations were quantified by interpolating from the generated standard curve (Figure S1).

#### 2.2.4 Metabolomics and Bioinformatics Analysis

The sample was subjected to three homogenization‐sonication cycles, left to stand for 1 h, centrifuged, and the supernatant was collected (Zhou et al. 2026). The final supernatants were submitted to Shanghai AQui Biotechnology Co. Ltd. for subsequent metabolomic analysis (Chicea et al. 2025). Relative abundance for the eight metabolites was expressed as normalized ion intensity obtained from UPLC‐MS‐based untargeted metabolomics after peak alignment and normalization.

#### 2.2.5 Network Pharmacology Analysis of Pertinent Target Pathways

The potential protein targets of the metabolic components of WGE‐EVLPs were predicted using the Swiss Target Prediction database (http://swisstargetprediction.ch/). The predicted targets were then deduplicated and consolidated to identify the common targets. The known therapeutic targets associated with ischemic stroke were retrieved from the GeneCards database (https://www.genecards.org/), and the OMIM database (https://www.omim.org/) (Li et al. 2026).

#### 2.2.6 High‐Performance Liquid Chromatography (HPLC)

Qualitative analysis of DGE‐EVLPs, WGE‐EVLPs, and GGE‐EVLPs was performed by HPLC‐DAD. The EVLPs samples were mixed with 95% methanol, subjected to three freeze–thaw cycles using liquid nitrogen and an ice bath, ultrasonicated, and centrifuged. The resulting supernatant was collected, dried under a stream of nitrogen, and reconstituted in methanol (Alhelwani et al. 2026).

#### 2.2.7 BV2 Cell Culture

BV2 cells were maintained in complete DMEM medium supplemented with 10% fetal bovine serum (FBS) at 37°C in a humidified atmosphere with 5% CO_2_ and were subcultured every 1 to 2 days based on their growth density (Yao and Fu 2020).

#### 2.2.8 Investigation of the Cytotoxic Effects of DGE‐EVLPs, WGE‐EVLPs, and GGE‐EVLPs on BV2 Cells

Following seeding in 96‐well plates at 5 × 10^4^ cells/mL, BV2 cells were allocated into control, model, and drug‐treated groups, the latter receiving compound concentrations from 5 to 320 μg/mL. After 24 h, the medium was replaced with compound‐containing medium for another 24 h. Then, 10 μL of CCK‐8 solution was added per well, and the plates were incubated at 37°C for 1–2 h before measuring the absorbance at 450 nm with a microplate reader for relative viability calculation (Wang et al. 2024).

#### 2.2.9 The Uptake Ability of DGE‐EVLPs, WGE‐EVLPs, and GGE‐EVLPs by BV2 Cells

##### 2.2.9.1 Flow Cytometry

Flow cytometry was employed to analyze the cellular uptake of DGE‐EVLPs, WGE‐EVLPs, and GGE‐EVLPs by BV2 cells. BV2 cells were seeded in 6‐well plates at a density of 1 × 10^6^ cells/mL and cultured for 24 h. The cells were then incubated with PKH67‐labeled EVLPs (160 μg/mL) for 12 h. After collection, the cells were centrifuged. The supernatant was carefully discarded, and the cell pellet was resuspended in phosphate‐buffered saline (PBS) in preparation for analysis by flow cytometry (Xie et al. 2025).

##### 2.2.9.2 High‐Content Imaging System

BV2 cells were seeded in 6‐well plates at 1 × 10^6^ cells/mL, cultured for 24 h, incubated with 10 μM DiI in the dark for 30 min for labeling, and then washed three times with PBS. Subsequently, the nuclei were stained with Hoechst reagent in the dark for 10 min. The cells were then co‐incubated with PKH67‐labeled WGE‐EVLPs (160 μg/mL). To exclude nonspecific fluorescence derived from PKH67 self‐aggregation or dye micelles, a dye‐only control was prepared by incubating PBS with PKH67 under the same labeling conditions used for WGE‐EVLPs, followed by the same purification/washing procedure. BV2 cells were incubated with this PBS + PKH67 preparation under identical conditions. Images were captured at 0 and 12 h to assess the uptake of WGE‐EVLPs by BV2 cells and to compare the differences between the drug‐treated and control groups (Xie et al. 2025).

#### 2.2.10 Role of DGE‐EVLPs, WGE‐EVLPs, and GGE‐EVLPs in OGD/R‐Induced BV2 Cell Injury

##### 2.2.10.1 Establishment of the OGD/R Model in BV2 Cells

BV2 cells were seeded in 96‐well plates at a density of 5 × 10^4^ cells/mL. After 12 h of culture, the medium was replaced with fresh medium containing DGE‐EVLPs, WGE‐EVLPs, or GGE‐EVLPs at concentrations of 20, 80, or 160 μg/mL for a 12 h treatment period. Following this, the medium was removed, and the cells were washed and subjected to the OGD/R insult. Briefly, the cells were incubated with glucose‐free DMEM medium and placed in a hypoxic chamber (5% CO_2_, 1% O_2_, 94% N_2_) at 37°C for 9 h of oxygen–glucose deprivation (OGD). This was followed by a 6‐h reoxygenation period by replacing the medium with standard complete medium under normoxic conditions (Weng et al. 2025).

##### 2.2.10.2 Cell Viability Assay

After being subjected to OGD/R, BV2 cells that had been seeded in 96‐well plates at a density of 5 × 10^4^ cells/mL were treated with 10 μL of CCK‐8 reagent per well. The plates were incubated at 37°C for 1 h, and the absorbance (*A*) at a wavelength of 450 nm was measured (Weng et al. 2025).

##### 2.2.10.3 Immunofluorescence Staining

BV2 cells were seeded in 24‐well plates at a density of 3 × 10^5^ cells/mL and treated with 160 μg/mL DGE‐EVLPs, WGE‐EVLPs, GGE‐EVLPs for 12 h followed by OGD/R. The cells were then fixed with 4% paraformaldehyde for 15 min, permeabilized with 0.1% Triton X‐100 for 15 min, and blocked with 5% BSA for 1 h. Thereafter, the cells were probed with rabbit anti‐CD86 monoclonal antibody (1:100) and rabbit anti‐CD206 polyclonal antibody (1:200) for 1.5 h. They were then treated with a fluorescein‐conjugated goat anti‐rabbit secondary antibody (1:200) at 37°C for 45 min under light‐shielded conditions. Finally, nuclear counterstaining was performed using 10 μM DAPI for 10 min in the absence of light. Images were acquired using a high‐content imaging system (Zhang et al. 2019).

##### 2.2.10.4 RT‐qPCR

Total RNA was extracted from cells using a rapid RNA extraction kit. cDNA synthesis was performed using the Evo M‐MLV Reverse Transcription Kit. To quantify gene expression, we performed reverse transcription quantitative PCR (RT‐qPCR) with the SYBR Green Pro Taq HS premixed kit (Li et al. 2021). The primer sequences utilized in this study are listed in Table S1.

#### 2.2.11 Biocompatibility Evaluation

##### 2.2.11.1 In Vitro Hemolysis Test

An in vitro hemolysis assay was performed using a 2% red blood cell suspension. The experimental setup included a negative control (normal saline), a positive control (distilled water), a solvent control (PBS), and three concentration groups (200, 500, and 1000 μg/mL) of DGE‐EVLPs, WGE‐EVLPs, and GGE‐EVLPs. Each group was tested in triplicate, with each tube containing 2.5 mL of the red blood cell suspension and an equal volume of the corresponding sample. After incubation at 37°C for 3 h, hemolysis was visually observed. Following water‐bath incubation, samples from each group were centrifuged at 800 × g for 5 min at room temperature. The resulting supernatant was collected, and its absorbance at 545 nm (A) was measured. The hemolysis rate was then determined using the following formula: Hemolysis rate (%) = (*A*_s_—*A*_n_)/(*A*_p_—*A*_n_) × 100%. A hemolysis rate below 5% was considered acceptable, indicating no significant hemolytic reaction. All tested samples showed hemolysis rates below 5%, complying with international standards and confirming their non‐hemolytic properties (Yang et al. 2019). (*A*_t_: treatment group; *A*_n_: negative control; *A*_p_: positive control).

##### 2.2.11.2 In Vivo Biocompatibility of DGE‐EVLPs, WGE‐EVLPs, and GGE‐EVLPs

Rats were randomly divided into experimental and blank control groups. Rats in the experimental groups were intragastrically administered DGE‐EVLPs, WGE‐EVLPs, or GGE‐EVLPs once daily for 14 consecutive days at a dose volume of 10 mL/kg. Control rats received the same volume of purified water. All animals exhibited normal feeding behavior. Prior to the final administration, the rats were fasted for 12 h. Following anesthesia with sodium pentobarbital, the control and treatment group rats (*n* = 3 per group) were euthanized. Major organs, including the heart, liver, spleen, lungs, and kidneys, were collected. After rinsing with physiological saline and fixation with chloral hydrate, the tissue samples were subjected to hematoxylin and eosin (H&E) staining. Histopathological examination and image acquisition were then performed under a microscope for analysis (Hwang et al. 2023).

#### 2.2.12 Statistical Analysis

All data are presented as mean ± standard deviation (SD). Normality and homogeneity of variance were assessed before group comparison. Comparisons among multiple groups were performed using one‐way ANOVA followed by Tukey's multiple‐comparison test for all pairwise comparisons or Dunnett's post hoc test when treatment groups were compared with a predefined control. When variances were unequal, Welch's ANOVA followed by Dunnett's T3 test was applied. A value of *p* < 0.05 was considered statistically significant.

## 3 Results

### 3.1 Physical Characterization of DGE‐EVLPs, WGE‐EVLPs, and GGE‐EVLPs

**FIGURE 1:**
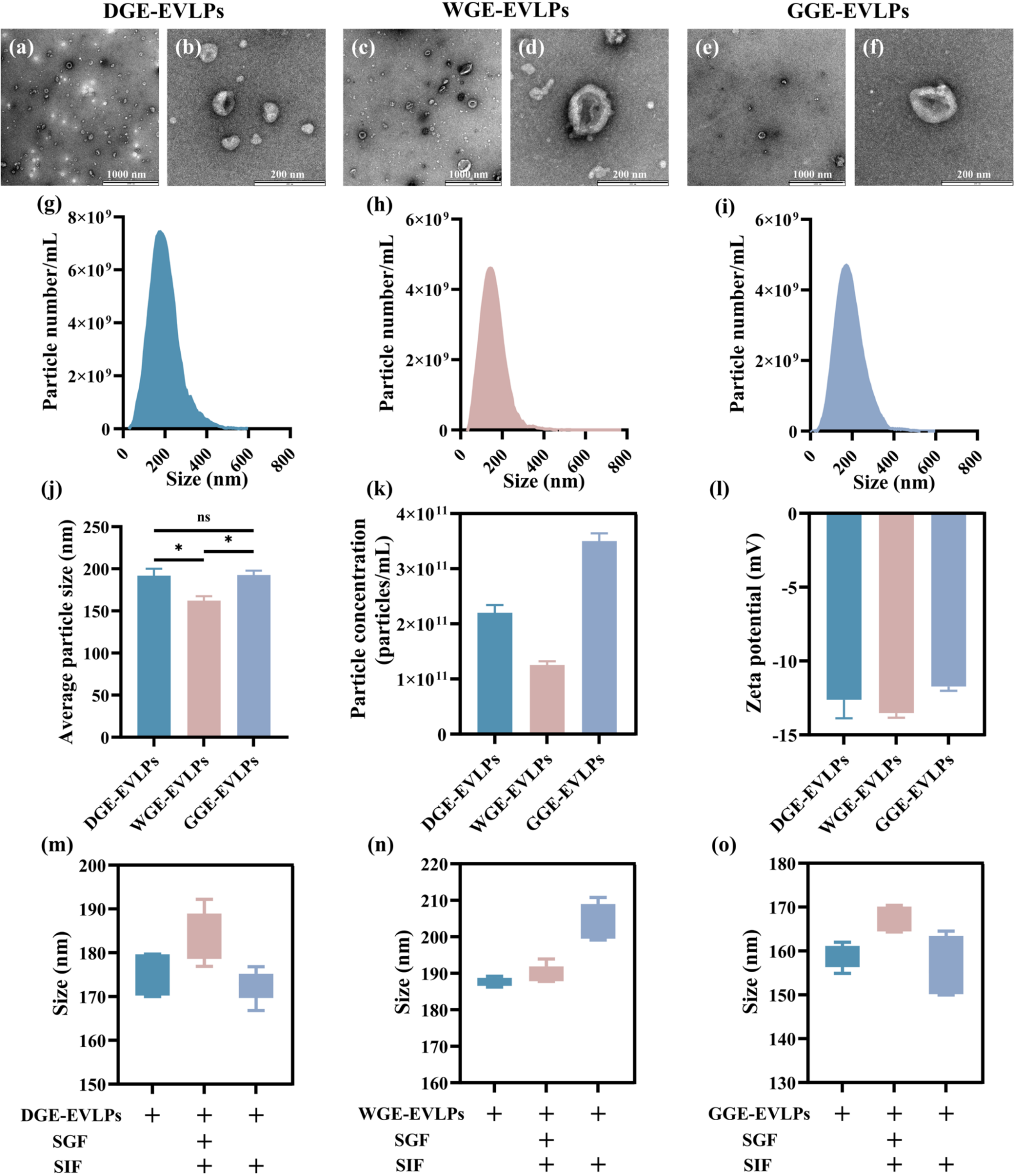
Characterization of DGE‐EVLPs, WGE‐EVLPs, GGE‐EVLPs. (a–f) TEM with scale bars of 1000 and 200 nm. (g–i) Particle size distribution of DGE‐EVLPs, WGE‐EVLPs, and GGE‐EVLPs. (j–l) Average particle size, particle concentration, and zeta potential of DGE‐EVLPs, WGE‐EVLPs, and GGE‐EVLPs (^ns^ *p* > 0.05, *p < 0.05; data are presented as mean ± SD; *n* = 3). (m–o) Stability of DGE‐EVLPs, WGE‐EVLPs, and GGE‐EVLPs in simulated gastric and intestinal fluids.

DGE, WGE, and GGE samples were firstly prepared from fresh rhizomes of GE following random grouping. After a series of pretreatment procedures, the corresponding EVLPs were isolated using a fully automated exosome extraction system and designated as DGE‐EVLPs, WGE‐EVLPs, and GGE‐EVLPs, respectively. The characteristic morphology of EVLPs was firstly examined. As shown in Figure 1a–f, TEM observations revealed that all three types of EVLPs exhibited a typical cup‐shaped morphology with integrity structure and uniform size. These results proved the existence of EVLPs in the three different kinds of processed GE. The particle distribution and concentration of DGE‐EVLPs, WGE‐EVLPs, and GGE‐EVLPs were investigated using NTA. The results demonstrated that the particle yields extracted from the same starting material mass were 2.3 × 10^11^ particles/mL, 1.2 × 10^11^ particles/mL, and 3.6 × 10^11^ particles/mL, respectively (Table S2). The average size of DGE‐EVLPs, WGE‐EVLPs, and GGE‐EVLPs were 197.7 nm, 158.3 nm, and 188.8 nm, respectively (Figure 1g–k; Table S2). All the vesicle size was under 200 nm. This result is consistent with the TEM findings and aligns with the characteristic size range of EVLPs. Notably, among the three groups, WGE‐EVLPs exhibited the smallest average particle size (158.3 nm), showing significant reductions compared to both DGE‐EVLPs (197.7 nm) and GGE‐EVLPs (188.8 nm) (*p* < 0.05), whereas no significant difference was observed between DGE‐EVLPs and GGE‐EVLPs (ns). This size difference may be closely related to the wine‐processing procedure employed during edible plants‐processing preparation. The processing procedure using yellow wine as a processing adjuvant, which may alter the osmotic pressure environment within the GE cells. And the yellow wine potentially promotes vesicle membrane contraction and structural remodeling, thereby leading to the formation of more and smaller vesicles. Recent research indicates that nanoparticle size is a critical factor affecting cellular uptake efficiency, with particles in the 100–200 nm range being more readily internalized by microglial cells via endocytosis. The gastrointestinal stability assay was used to evaluate whether GE‐EVLPs maintained their nanoscale dispersion after exposure to simulated gastric and intestinal fluids. Rather than showing a monotonic trend, the expected result was the absence of marked aggregation or collapse. The three GE‐EVLPs retained a generally stable nanoscale size distribution under the tested simulated digestive conditions (Figure 1m–o; Tan et al. 2025).

Zeta potential analysis results (Figure 1l) showed values of −12.62 ± 1.23 mV, −13.53 ± 0.31 mV, and −11.74 ± 0.29 mV for DGE‐EVLPs, WGE‐EVLPs, and GGE‐EVLPs, respectively. The observed negative zeta potentials are consistent with the surface charge characteristics of a phospholipid bilayer. This structure also confers upon them the potential to serve as functional carriers for the targeted delivery of active ingredients. Among all the data, WGE‐EVLPs exhibited the highest absolute zeta potential value (−13.53 ± 0.31 mV), which was significantly higher than that of DGE‐EVLPs and GGE‐EVLPs (*p < 0.05), indicating the superior colloidal stability of WGE‐EVLPs. This property is conducive to prolonging their circulation time in vivo and thus enhancing bioavailability.

### 3.2 Compositional Analysis of DGE‐EVLPs, WGE‐EVLPs, and GGE‐EVLPs

**FIGURE 2:**
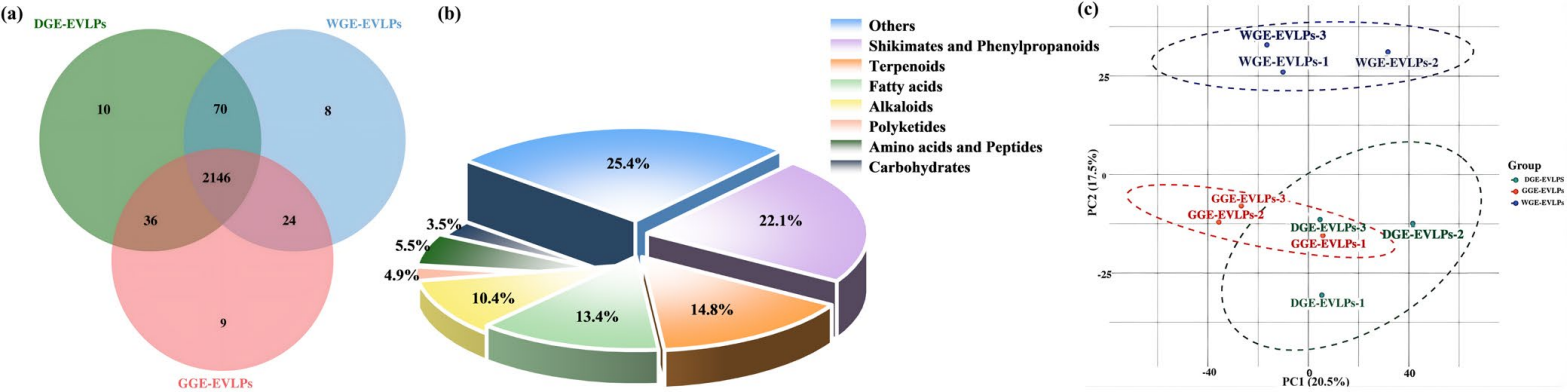
(a–c) Venn diagram, compound distribution, and PCA score plot for DGE‐EVLPs, WGE‐EVLPs, and GGE‐EVLPs.

**FIGURE 3:**
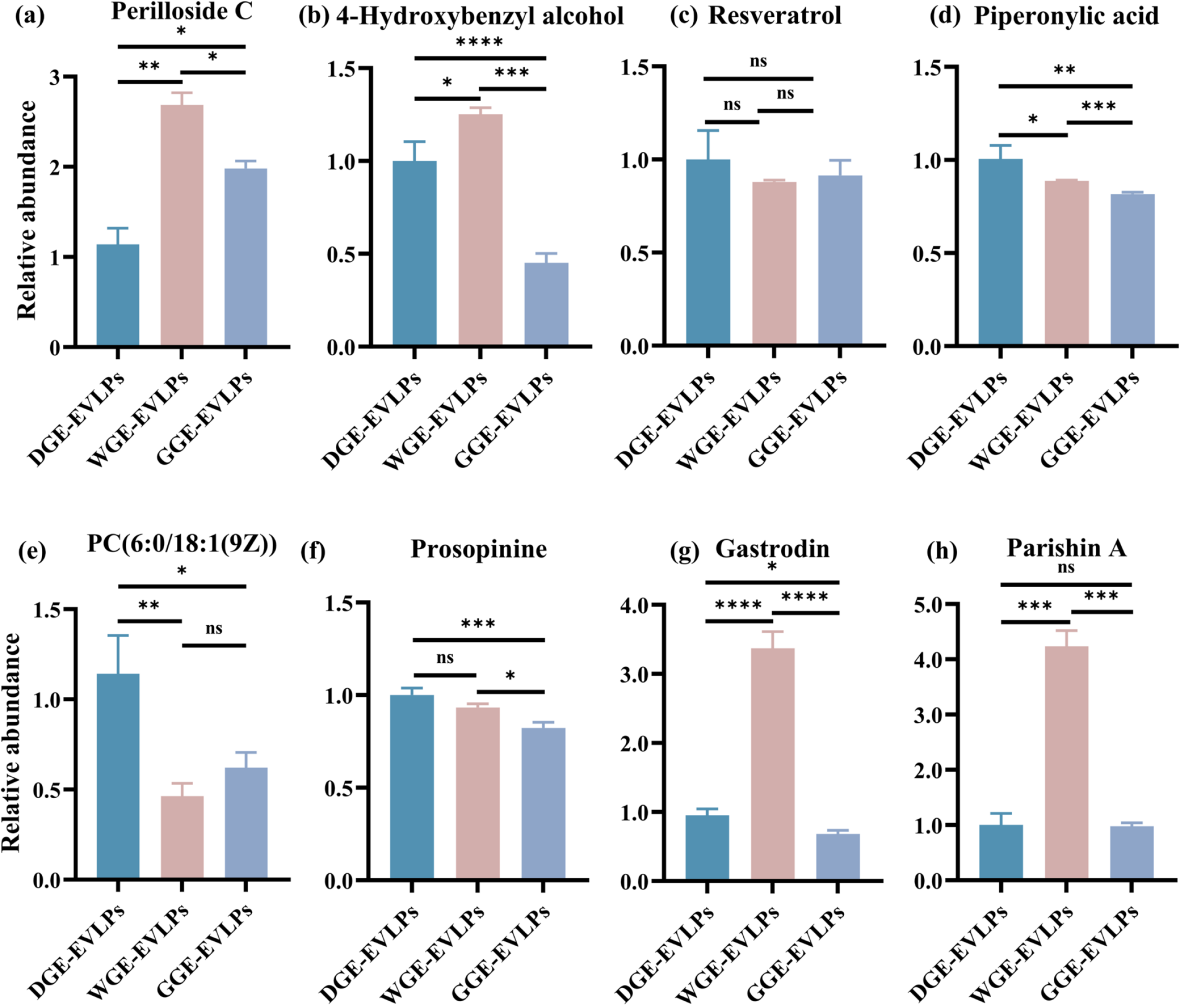
Relative abundance of eight metabolites in DGE‐EVLPs, WGE‐EVLPs, and GGE‐EVLPs: (a) Perilloside C, (b) 4‐Hydroxybenzyl alcohol, (c) Resveratrol, (d) Piperonylic acid, (e) PC(6:0/18:1(9Z)), (f) Prosopinine, (g) Gastrodin, and (h) Parishin A. ^ns^ *p* > 0.05, *p < 0.05, **p < 0.01, ***p < 0.001, ****p < 0.0001. Data are presented as mean ± SD (*n* = 3).

Edible plant derived EVLPs are nanoscale structures secreted by plant cells, enveloped by a phospholipid bilayer. They can carry various active components such as proteins, nucleic acids, and metabolites, and are involved in metabolic regulation within the plant. To identify the active components and difference, we performed untargeted metabolomic sequencing on DGE‐EVLPs, WGE‐EVLPs, and GGE‐EVLPs, respectively. The three EVLPs samples were analyzed by UPLC‐MS. The total ion chromatograms (TICs) of three EVLPs extracts acquired in both positive and negative ionization modes are presented in Figures S2 and S3. As shown in Figure 2a, the experimental results indicated that DGE‐EVLPs, WGE‐EVLPs, and GGE‐EVLPs shared a total of 2146 common metabolic components. The major classes of compounds identified in the three types of GE derived EVLPs included coumarins and phenylpropanoids, terpenoids, fatty acids, and alkaloids (Figure 2b). The PCA plot (Figure 2c) showed obvious differences among the WGE‐EVLPs with DGE‐EVLPs and GGE‐EVLPs. KEGG pathway analysis revealed that different GE‐derived EVLPs can target distinct metabolic pathways. These differential regulatory effects may subsequently translate into their specific physiological regulatory functions (Figure S4a). The Z‐score analysis of major differential metabolites among DGE‐EVLPs, WGE‐EVLPs, and GGE‐EVLPs was compared in Figure S4b. However, this observation remains dependent on the specific molecular features identified and requires further validation. In addition to the visualization of clusters made possible by PCA, univariate analyzes were processed to complete the investigation with a factor of interest focused on the type of GE derived EVLPs. Figure 3 depicts the variability in some obvious differential metabolite distribution among DGE‐EVLPs, WGE‐EVLPs, and GGE‐EVLPs. Typically, Perilloside C may confer potential protective effects against neuronal damage induced by cerebral ischemia in diabetes or chronic hyperglycemia, likely through inhibiting AR, reducing sorbitol accumulation, and mitigating downstream oxidative stress (Tomoyuki et al. 1995). 4‐Hydroxybenzyl alcohol protects against cerebral ischemia–reperfusion injury in rats by reducing blood–brain barrier permeability, thereby exerting a blood–brain barrier‐protective effect (Wen et al. 2016). Resveratrol, a natural phytoalexin, exerts anti‐inflammatory, antioxidant, anti‐apoptotic, and edema‐reducing effects and has been reported to improve ischemic stroke outcomes (Owjfard et al. 2024; Yu and Hui 2024). Gastrodin exerts robust antioxidant, anti‐inflammatory, and blood–brain barrier–protective properties, thereby preventing and attenuating neuronal damage elicited by cerebral ischemia/reperfusion injury (Qin et al. 2025). The natural small‐molecule Parishin A modulates inflammatory responses by driving M2 macrophage polarization via the JAK/STAT1 pathway (Zhu et al. 2023). The higher abundance of these metabolites in WGE‐EVLPs than DGE‐EVLPs and GGE‐EVLPs may influence their function and application. Besides, our group analyzed the chemical compositions of raw and processed GE, which led to the identification of seven differential metabolites: gastrodin, *p*‐hydroxybenzyl alcohol, *p*‐hydroxybenzaldehyde, and parishins A, B, C, and E in a previous study (Su et al. 2024). Building upon this foundation, the current study established a HPLC method for the detection of these specific compounds. This method was subsequently applied to analyze the presence of the corresponding components in DGE‐EVLPs, WGE‐EVLPs, and GGE‐EVLPs. The results demonstrated that all three types of EVLPs contained six of the seven targeted components: gastrodin, *p*‐hydroxybenzyl alcohol, and parishins A, B, C, and E. In contrast, *p*‐hydroxybenzaldehyde was detected only in minor quantities in DGE‐EVLPs. Integrated with the results from untargeted metabolomics and HPLC‐DAD detection of seven characteristic metabolic compounds (Figure S5), the small‐molecule active components within the EVLPs derived from processed GE were largely consistent with those of the processed plants themselves. This suggests that EVLPs possess the potential to carry various active components from GE and mediate multiple biological activities.

**FIGURE 4:**
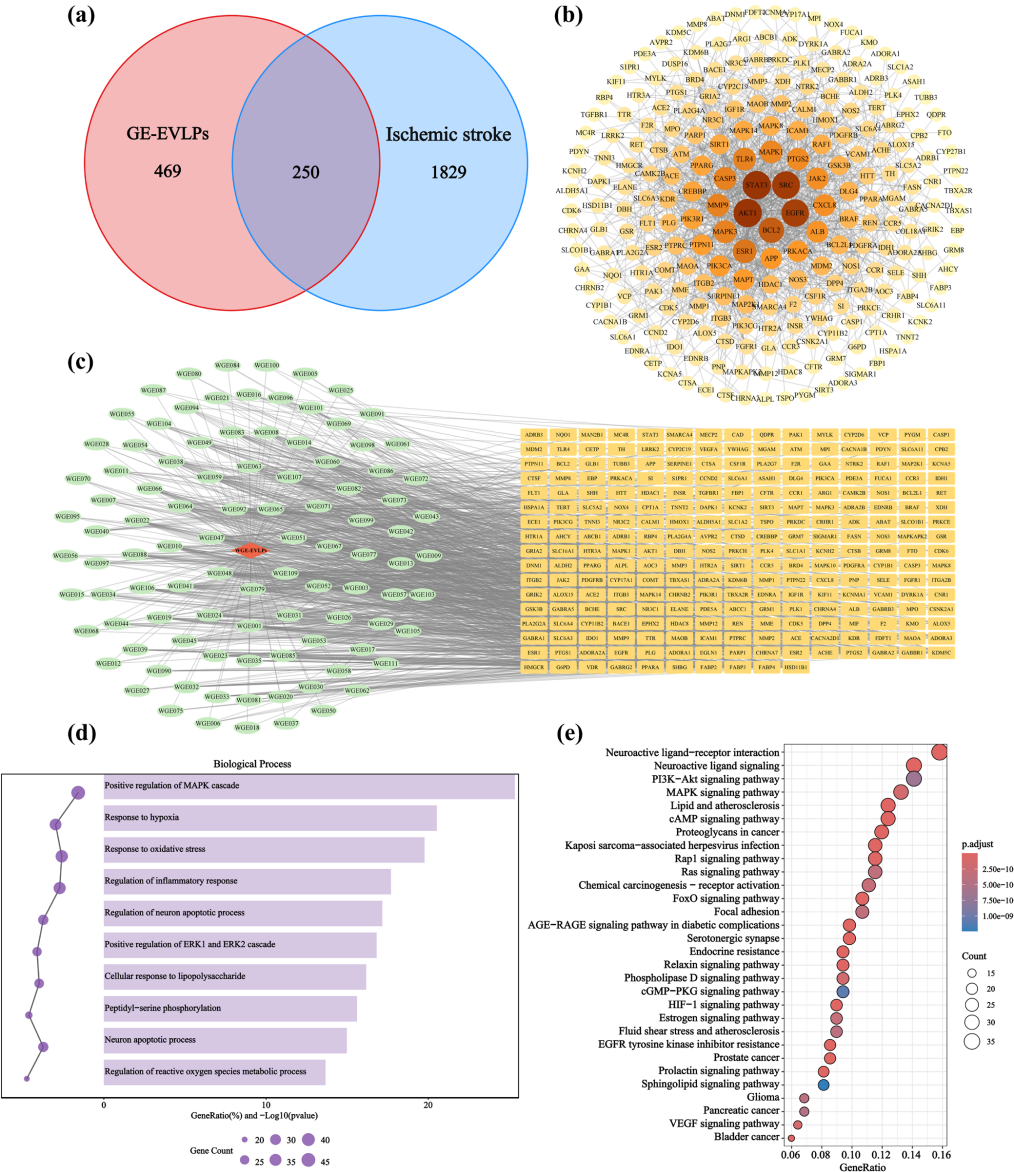
(a) Intersection of potential target genes between GE‐EVLPs and ischemic stroke. (b, c) Comparative analysis of the potential target gene networks for GE‐EVLPs and ischemic stroke. (d) BP in GO pathway enrichment map of potential target genes. (e) KEGG pathway enrichment map of potential target genes.

To elucidate the potential neuroprotection effects of GE‐EVLPs as a functional ingredient, we further investigated the highly abundant compounds in GE‐EVLPs. An intersection analysis with 2079 disease targets from the GeneCards and OMIM databases revealed 250 overlapping targets, accounting for 9.8% of the disease‐associated targets (Figure 4a). To prioritize functionally critical nodes among these targets, a protein–protein interaction (PPI) network was constructed and visualized in Cytoscape, with node color intensity reflecting topological importance. As shown in Figure 4b, STAT3, SRC, JUN, TP53, AKT1, and MAPK1 exhibited the highest degree values, identifying them as hub targets likely mediating the neuroprotective effects of GE‐EVLPs. To dissect the polypharmacological basis of this action, a compound‐target network was subsequently established (Figure 4c). This architecture underscores the multi‐component, multi‐target synergistic nature of GE‐EVLPs, whereby diverse chemical constituents collaboratively regulate key nodes in neuroinflammatory and anti‐apoptotic pathways. We performed a comprehensive Gene Ontology (GO) enrichment analysis on the core targets. The analysis covered three major domains: Biological Process (BP, Figure 4d), Cellular Component (CC, Figure S6), and Molecular Function (MF, Figure S7). Kyoto Encyclopedia of Genes and Genomes (KEGG) pathway enrichment analysis further revealed that the targets were significantly associated with neuroactive ligand‐receptor interaction pathways (Figure 4e).

**FIGURE 5:**
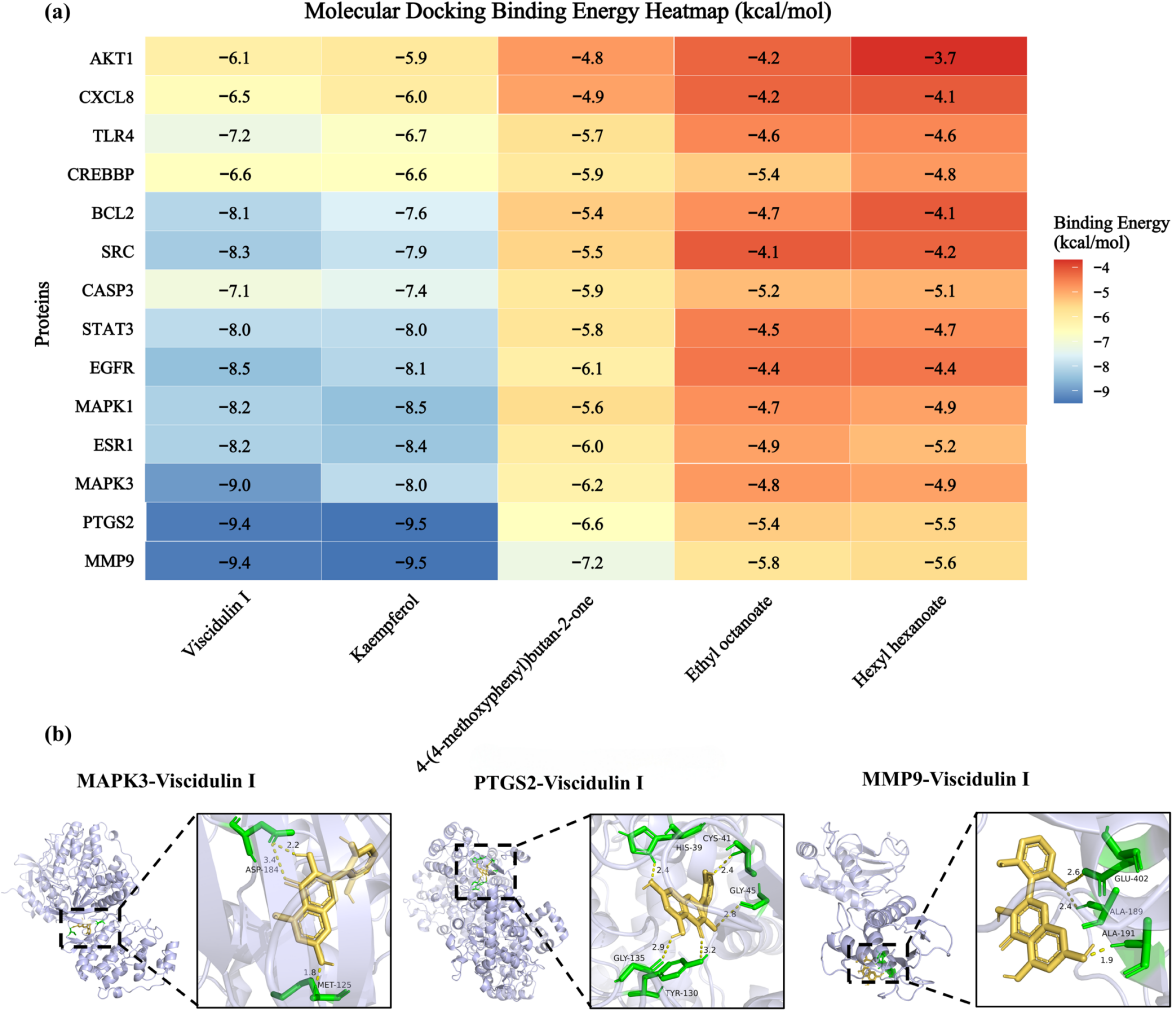
(a) Docking scores between 14 proteins and the 5 major compounds of GE‐EVLPs. (b) Schematic illustration of the molecular docking between Viscidulin I and the target proteins.

The binding capacity of GE‐EVLPs to therapeutic targets for ischemic stroke, such as MAPK3, PTGS2, and MMP9, was investigated using computational molecular docking. In the AutoDock Vina paradigm, more negative docking scores denote progressively stronger ligand–receptor interactions and greater thermodynamic stability of the resulting complex (Nigdelioglu Dolanbay and Aslim 2025). Consistent with established benchmarking cut‐offs, binding free energies ≤ −4.25 kcal/mol were taken to indicate robust target engagement by the bioactive constituents, whereas energies ≤ −9.0 kcal/mol were regarded as representative of the strongest binding tier (Figure 5a) (He et al. 2025). Highly expressed compounds including Viscidulin I and kaempferol consistently demonstrated strong binding affinity toward key targets. Notably, Viscidulin I exhibited interaction energies below −9 kcal/mol with all three therapeutic targets examined, suggesting that this compound fits well into the active pocket of the targets and exhibits high receptor–ligand affinity (Figure 5b; Figure S8).

The BCA assay yielded protein concentrations of 4.8 μg/μL for DGE‐EVLPs, 11.0 μg/μL for WGE‐EVLPs, and 6.2 μg/μL for GGE‐EVLPs, as presented in Figure S9. Based on these measurements, the particle‐to‐protein ratios were calculated to be 4.8 × 10^9^ particles/μg for DGE‐EVLPs, 1.1 × 10^9^ particles/μg for WGE‐EVLPs, and 5.8 × 10^9^ particles/μg for GGE‐EVLPs. These ratios, all falling within the same order of magnitude, indicate that the three types of EVLPs are comparable in terms of both their particle concentration and protein content.

### 3.3 Biocompatibility Evaluation

**FIGURE 6:**
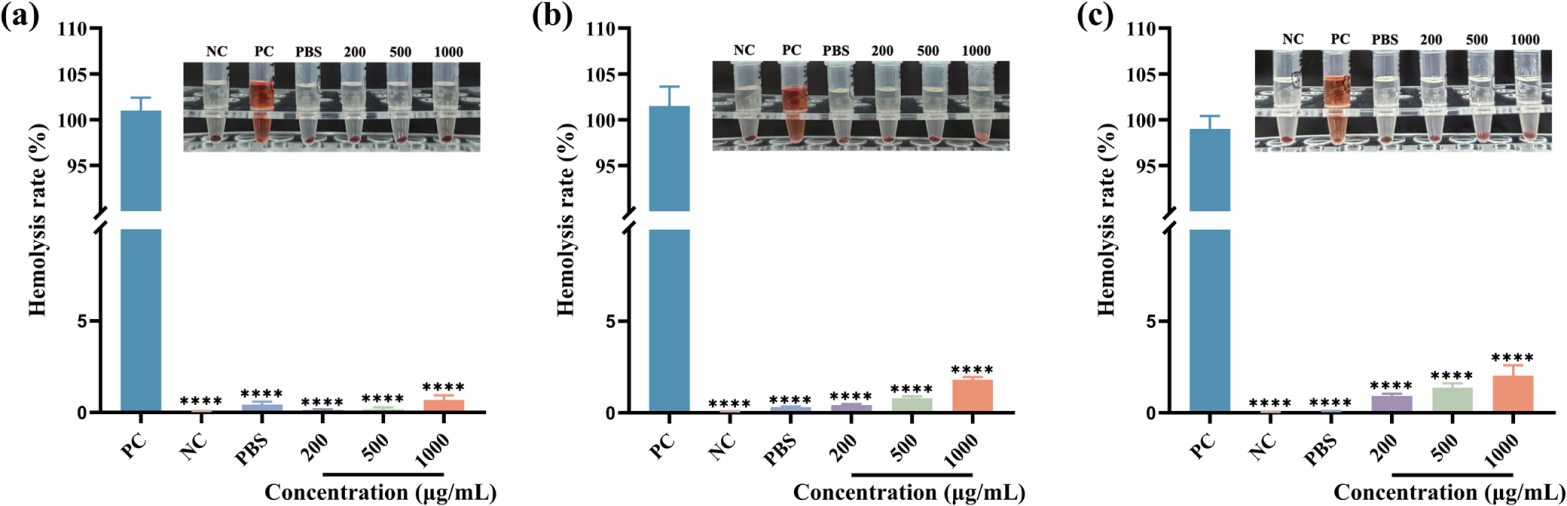
Results and photographs of the hemocompatibility assay for (a) DGE‐EVLPs, (b) WGE‐EVLPs, and (c) GGE‐EVLPs (****p < 0.0001 vs. PC; data are presented as mean ± SD; *n* = 3).

The hemolysis assay serves as a key method for evaluating the biocompatibility and blood safety of food‐derived ingredients, providing critical in vitro evidence for their biosafety assessment. In this study, hemolysis tests were firstly conducted on DGE‐EVLPs, WGE‐EVLPs, and GGE‐EVLPs. The experimental results showed that the supernatant in the negative control group (NC, normal saline group) was colorless and clear, with no hemolysis observed. In contrast, the supernatant in the positive control group (PC, distilled water group) was red and transparent, indicating obvious hemolysis. The supernatants from both the PBS and all concentration groups of the three EVLPs were clear and transparent, which indicated no significant hemolysis was detected. After centrifugation, the supernatants from all groups were transferred to a 96‐well plate, and the absorbance was measured at 545 nm to quantify the hemolysis situation. The degree of hemolysis was calculated relative to the positive control group, which was set as 100% hemolysis. As shown in Figure 6a–c, DGE‐EVLPs, WGE‐EVLPs, and GGE‐EVLPs at all tested concentrations (200–1000 μg/mL) exhibited negligible hemolysis rates (< 5%), comparable to the NC and PBS groups and well below the 5% safety threshold defined by the ISO 10993‐5 standard (Yang et al. 2019). Compared with the PC group, all EVLPs‐treated groups displayed extremely significant reductions in hemolysis rates (****p < 0.0001), confirming the absence of hemolytic toxicity. Notably, the hemolysis rate of WGE‐EVLPs remained as low as 1.81% even at the highest concentration of 1000 μg/mL, indicating that the wine‐processing technique did not introduce hemolytic contaminants or compromise hematological safety (Yang et al. 2019).

Furthermore, a 14‐day oral gavage study was performed to evaluate systemic biosafety. H&E staining revealed no significant histopathological lesions in the heart, kidney, spleen, lung, or liver of rats treated with DGE‐EVLPs, WGE‐EVLPs, or GGE‐EVLPs relative to controls (Figure S10). Microscopic examination confirmed normal tissue architectures across all organs: cardiac myofibers were regularly arranged with distinct striations; renal glomeruli and tubular epithelium remained intact with clear corticomedullary differentiation; splenic white and red pulp were well defined without lymphoid depletion or hemorrhage; pulmonary alveolar structures were preserved without edema, hemorrhage, or inflammatory infiltration; and hepatic hepatocytes exhibited normal polygonal morphology with central nuclei and intact sinusoids. These results indicate that 14‐day repeated oral administration of these GE‐EVLPs did not induce detectable pathological changes in rat visceral organs.

### 3.4 Cellular Uptake of DGE‐EVLPs, WGE‐EVLPs, and GGE‐EVLPs by BV2 Cells

**FIGURE 7:**
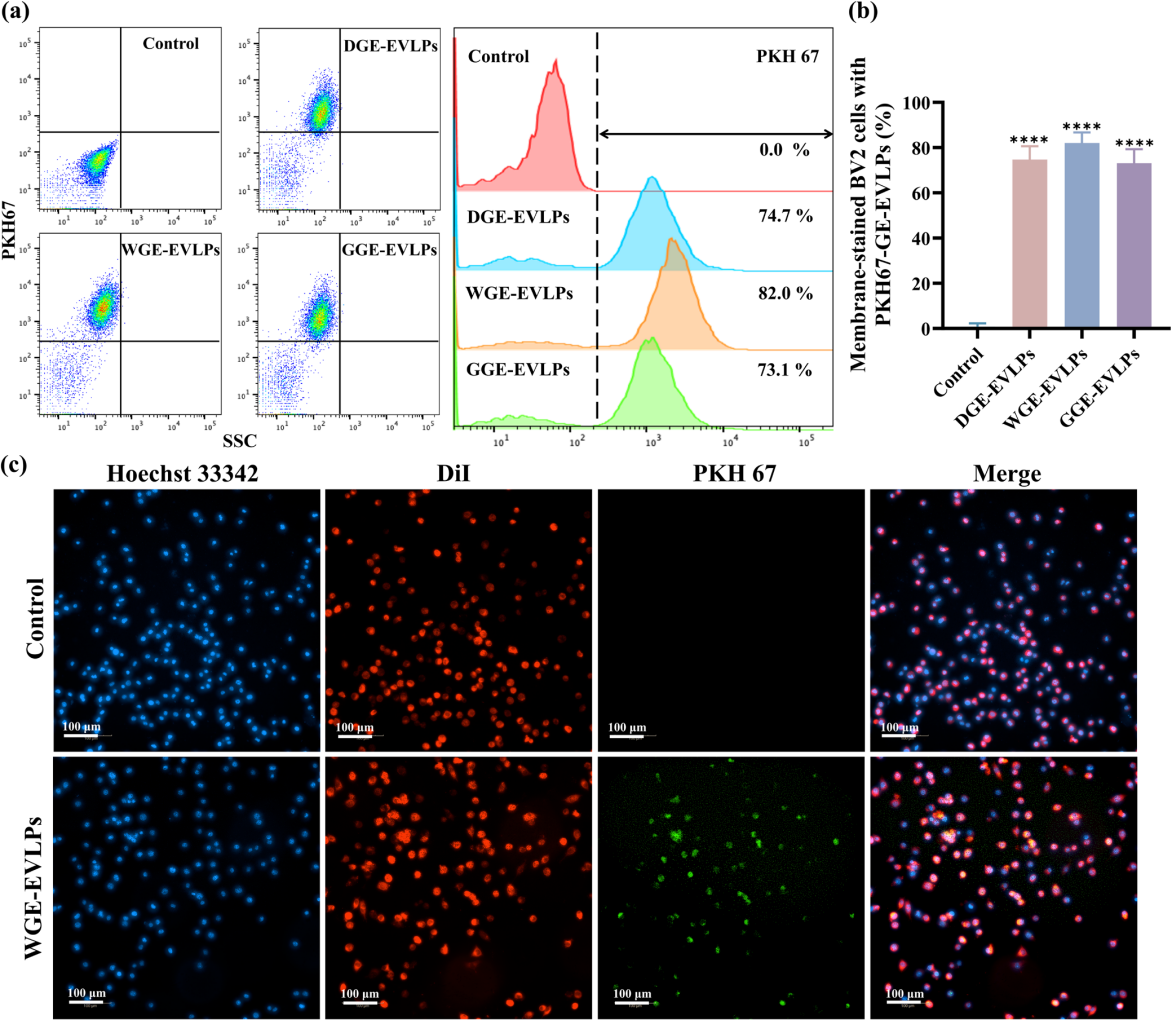
Uptake of EVLPs by BV2 cells. Uptake of PKH67‐labeled EVLPs by BV2 cells by (a) flow cytometry, (b) corresponding histogram of cellular uptake of PKH67‐labeled EVLPs in BV2 cells, and (c) fluorescence imaging (****p < 0.0001 vs. Control; data are presented as mean ± SD; *n* = 3).

The efficiency of cellular uptake directly influences the subsequent effects of EVLPs on cellular function and survival. To enable statistical comparison of BV2 cell uptake across different EVLPs preparations, PKH67‐fluorescently labeled DGE‐EVLPs, WGE‐EVLPs, and GGE‐EVLPs (160 μg/mL) were subjected to uptake assays with an incubation time of 12 h (*n* = 3). Flow cytometry analysis (Figure 7a) revealed a marked fluorescent shift in the PKH67 channel for all EVLPs‐treated groups compared to the untreated control. Quantitative analysis showed that the proportions of PKH67‐positive BV2 cells were 74.7% for DGE‐EVLPs, 82.0% for WGE‐EVLPs, and 73.1% for GGE‐EVLPs, all of which were extremely significantly higher than that of the control group (****p < 0.0001, Figure 7b). Notably, among the three preparations, WGE‐EVLPs displayed the highest cellular uptake efficiency (*p < 0.05).

Subsequently, the cellular uptake was further examined using a fluorescence imaging system, with WGE‐EVLPs as a representative example. As shown in Figure 7c, blue (DAPI, cell nuclear dye) and red (DiI, cell membrane dye) fluorescence indicated cell number and distribution, respectively. The absence of nuclear overlap and clear cell membrane boundaries demonstrated that the cells grew into a tight, confluent monolayer with good viability after a 12 h culture. Compared to the blank control group (without WGE‐EVLPs), distinct green fluorescence signals (PKH67) were observed surrounding the nuclei in the WGE‐EVLPs‐treated group. For the dye‐only negative control, PBS without EVLPs was incubated with PKH67 under the same labeling conditions as EVLPs samples, quenched and washed using the same procedure, and then added to BV2 cells. The absence of obvious green fluorescence in this control confirmed that the fluorescence signal observed in the EVLPs‐treated group was not attributable to free PKH67 dye micelles (Figure S11). Cellular uptake experiments collectively demonstrate that EVLPs are internalized by cells while maintaining their structural integrity, establishing a foundation for their application as delivery vehicles for dietary components.

**FIGURE 8:**
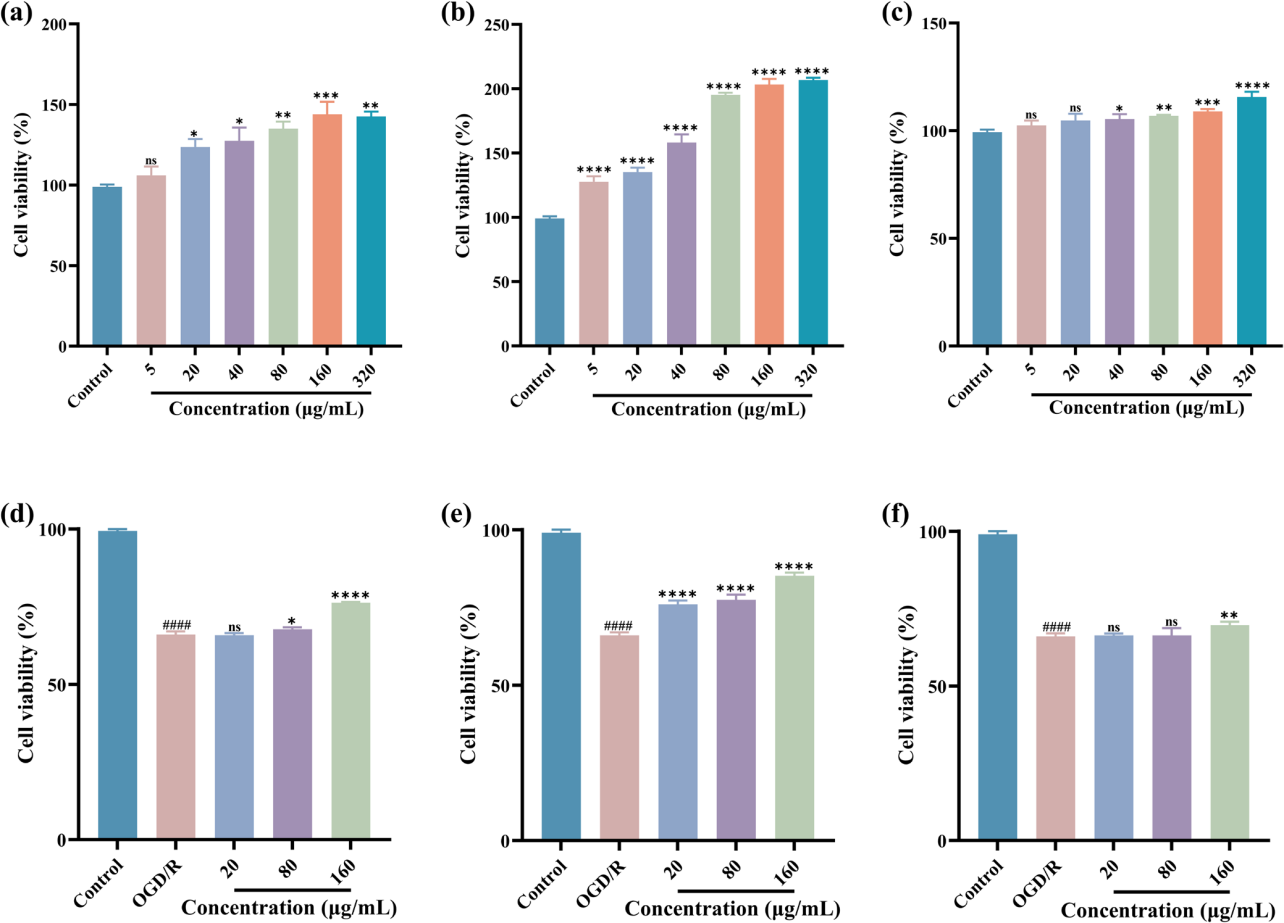
Evaluation of cytotoxicity and protective effects of DGE‐EVLPs, WGE‐EVLPs, and GGE‐EVLPs against OGD/R‐induced injury in BV2 cells. (a–c) Proliferative capacity of BV2 cells treated with DGE‐EVLPs, WGE‐EVLPs, and GGE‐EVLPs. ^ns^ *p* > 0.05, *p < 0.05, **p < 0.01, ***p < 0.001, ****p < 0.0001 versus Control. (d–f) OGD/R‐induced injury in BV2 cells. ^####^ *p* < 0.0001 versus Control. ^ns^ *p* > 0.05, *p < 0.05, **p < 0.01, ***p < 0.001, ****p < 0.0001 versus OGD/R (*n* = 3, mean ± SD).

The cytotoxic and proliferative effects of DGE‐EVLPs, WGE‐EVLPs, and GGE‐EVLPs on BV2 cells were evaluated using the CCK‐8 assay. In this system, absorbance values exceeding those of the untreated control denote elevated metabolic activity, which, under non‐cytotoxic conditions, corresponds to enhanced proliferative capacity. Cells were exposed to concentrations ranging from 5 to 320.0 μg/mL, with complete culture medium serving as the control. Following 24‐h incubation at 37°C (Figure 8a–c), all three GE‐derived EVLPs significantly promoted BV2 cell proliferation relative to the control group (*p < 0.05). At 160 μg/mL, the relative proliferation rates of DGE‐EVLPs‐, WGE‐EVLPs‐, and GGE‐EVLPs‐treated cells reached 143.90%, 203.33%, and 109%, respectively, with WGE‐EVLPs showing significantly higher proliferation than both DGE‐EVLPs and GGE‐EVLPs (*p < 0.05), confirming the absence of cytotoxicity across the entire tested concentration range. Notably, WGE‐EVLPs at 320 μg/mL further elevated proliferative activity to 206.75%, implying that these edible plant‐derived EVLPs may harbor endogenous nutritive properties capable of stimulating cell growth. This proliferation‐enhancing effect is particularly pertinent to neurological health, wherein preserving microglial function is essential for maintaining neural homeostasis (Li et al. 2021). The robust capacity of these EVLPs to sustain cell proliferation underscores their promise as functional ingredients in health‐oriented food applications. Moreover, GE‐derived EVLPs may act synergistically: they not only possess intrinsic nutritional and bioactive functions but also serve as nanocarriers for exogenous functional ingredients, thereby offering a dual strategy for augmenting health benefits.

### 3.5 Protective Benefits of DGE‐EVLPs, WGE‐EVLPs, and GGE‐EVLPs in BV2 Cells Subjected to OGD/R

To evaluate the neuroprotection functions of three GE derived EVLPs, an oxygen–glucose deprivation/reoxygenation (OGD/R) model was established in BV2 cells. This study investigated their role in supporting cellular functional integrity, and a systematic comparison was conducted on the interventional effects of DGE‐EVLPs, WGE‐EVLPs, and GGE‐EVLPs. As shown in Figure 8d–f, compared to the normal control group, cell viability in the model group was significantly decreased (**p < 0.01), indicating the successful induction of the OGD/R model. Treatment with WGE‐EVLPs increased BV2 cells CCK‐8 metabolic activity after OGD/R in a concentration‐related manner, whereas GGE‐EVLPs produced a significant improvement only at the effective tested concentration (*p < 0.05). Compared with the model group, the BV2‐cell viability was increased 10.21%, (*p < 0.05), 19.15% (***p < 0.001) and 3.67% (ns, *p* > 0.05) when DGE‐EVLPs, WGE‐EVLPs, and GGE‐EVLPs were added with a concentration of 160 μg/mL. Specifically, WGE‐EVLPs at concentrations of 20, 80, and 160 μg/mL effectively enhanced cell survival. Notably, WGE‐EVLPs exhibited a significant protective effect even at the low concentration of 20 μg/mL (*p < 0.05). The most potent effect was observed at 160 μg/mL (***p < 0.001), where WGE‐EVLPs increased cell viability from 66.00% in the model group to 85.15%. These results indicate that, among the three GE‐derived EVLPs, WGE‐EVLPs may possess superior potential for supporting neurological health.

**FIGURE 9:**
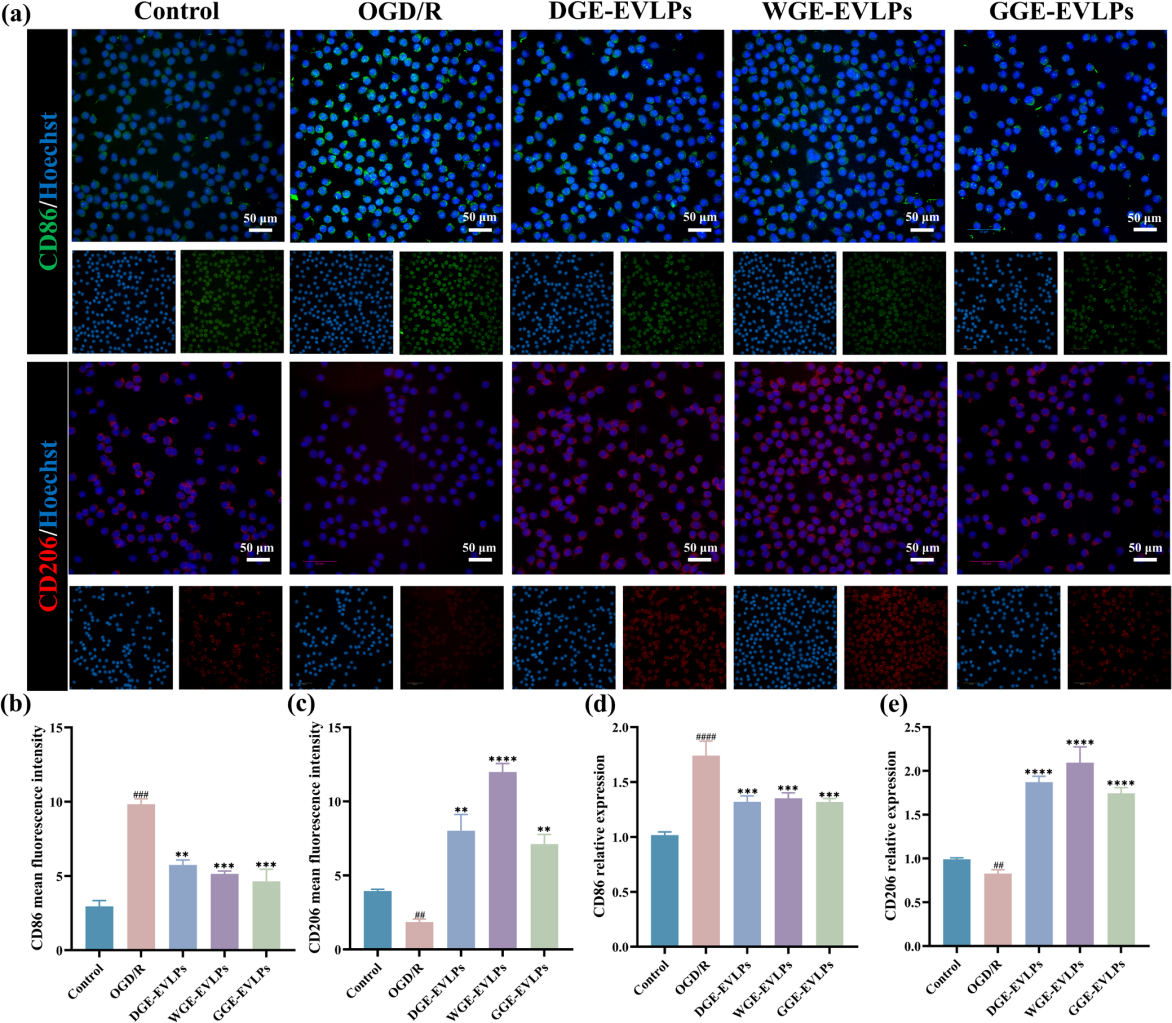
Effects of DGE‐EVLPs, WGE‐EVLPs, and GGE‐EVLPs on the expression of CD86 and CD206 in BV2 cells. (a–c) Immunofluorescence staining (scale bar of 50 μm) and (d, e) RT‐qPCR. ^##^ *p* < 0.01, ^###^ *p* < 0.001, ^####^ *p* < 0.0001 versus Control. **p < 0.01, ***p < 0.001, ****p < 0.0001 versus OGD/R (*n* = 3, mean ± SD).

To further investigate the effect of DGE‐EVLPs, WGE‐EVLPs, and GGE‐EVLPs, the influence on polarization procedure was examined by immunofluorescence staining and RT‐qPCR. As shown in Figure 9a–c, the immunofluorescence results revealed that compared with the control group, the expression of the M1 phenotype marker CD86 was significantly increased in the model group (^##^ *p* < 0.01). However, all three EVLPs treatment groups showed a marked decrease in CD86 fluorescence intensity relative to the model group (*p < 0.05), suggesting an inhibitory effect of GE‐derived EVLPs on microglial M1 polarization. On the other hand, there was no significant difference in the fluorescence intensity of the M2 phenotype marker CD206 between the model group and the control group. Furthermore, CD206 protein expression was significantly enhanced in the three GE‐derived EVLPs compared to the model group, especially the WGE‐EVLPs (160 μg/mL) treatment group (***p < 0.001). The results of RT‐qPCR indicated that after incubation for 24 h, the expression levels of CD86 in the M1 phenotype marker decreased in the DGE‐EVLPs, WGE‐EVLPs, and GGE‐EVLPs, whereas the expression levels of CD206 in the M2 phenotype marker increased (*p < 0.05) (Figure 9d,e).

## 4 Discussion

The processing of edible plants constitutes a central practice designed to improve their nutritional and health benefits, reduce potential adverse constituents, and optimize their stability and storage characteristics, thus securing their reliability as food ingredients (Hao et al. 2024; Zhou et al. 2023). Consequently, this longstanding and sophisticated practice is critical for unraveling the scientific rationale behind the advanced processing of food‐grade plant materials. However, whether processed food‐medicine homologous plants contain EVLPs, and the influence on their physicochemical properties and potential applications, has not been systematically investigated. In this study, for the first time, we targeted the applied processed products of GE including DGE, WGE and GGE. Successful isolation confirmed the presence of EVLPs from all three processing methods. This study aims to reveal whether processing methods can significantly influence the physicochemical properties and biological activities of GE‐derived EVLPs as follows: (1) the physical characterization results, DGE‐EVLPs, WGE‐EVLPs, and GGE‐EVLPs all exhibited typical characteristics of EVLPs: an intact bilayer membrane structure, a particle size distribution within 200 nm, and a negatively charged surface (Liu et al. 2024; Zhao et al. 2024). (2) The content information analysis probed the existence of protein and various active small molecule components in the three kinds of GE derived EVLPs. (3) The biosafety evaluation was conducted in three parts: cytotoxicity on BV2 cells, hemolysis, and biocompatibility in rats. All the biosafety results exhibit the potential for applying them as natural nanocarriers (Liang et al. 2025). (4) All three GE‐derived EVLPs help preserve the functional integrity of BV2 cells in the OGD/R model (Weng et al. 2025).

Compared with crude GE preparations, GE‐derived EVLPs represent a nanoscale lipid‐bilayer system capable of carrying endogenous metabolites, proteins, lipids, and nucleic‐acid‐like cargos (Hao et al. 2025). This vesicular structure may protect bioactive constituents during gastrointestinal exposure, facilitate cellular uptake, and provide a naturally sourced food‐grade delivery platform. Therefore, GE‐EVLPs should not be viewed simply as a replacement for GE but as a distinct functional fraction that may help explain processing‐related changes in bioavailability and biological activity.

Among them, WGE‐EVLPs, obtained via wine‐processing technology, exhibit notably superior attributes, including a small particle size (158.3 nm), high cellular uptake efficiency, a unique composition, and a significant protective effect in the OGD/R model. These characteristics are closely linked to their underlying health‐promoting mechanisms: high bioavailability ensures potent bioactivity even at low concentrations (20 μg/mL); suitable nanoscale properties enhance delivery efficiency; distinct composition may contribute to specific protective functions; and health‐modulating effects at the cellular level by regulating immune‐related markers (Fu et al. 2025).

Although this study systematically compared EVLPs derived from DGE, WGE, and GGE, several limitations should be acknowledged. First, yellow wine and ginger juice may introduce their own vesicle‐like nanoparticles or soluble constituents during processing, which could contribute to the composition and biological effects of WGE‐EVLPs and GGE‐EVLPs. Therefore, although the present results reflect the EVLPs‐containing fractions of traditionally processed GE products, they cannot fully distinguish GE‐derived EVLPs from potential adjuvant‐derived vesicular or soluble contributions. Future studies will use EVLPs‐depleted yellow wine and ginger juice, prepared by ultracentrifugation and/or membrane filtration, as processing controls to clarify the contribution of processing adjuvants. Second, plant EVLPs are generally considered to include vesicles secreted into the apoplast through multivesicular body/plasma‐membrane‐related pathways and other unconventional secretion routes. However, in the present study, GE‐EVLPs were isolated from total processed tuber homogenates rather than apoplastic washing fluid. Thus, their precise subcellular origin cannot be definitively assigned. Third, neuroprotection was assessed chiefly in an OGD/R‐challenged BV2 cell model, with only subchronic oral biosafety evaluations to support systemic translational potential; in vivo ischemia models, biodistribution, pharmacokinetics, and long‐term safety studies remain necessary. Fourth, the relationship between specific EVLPs cargo molecules and the observed biological effects remains correlative. Future work should include EVLP‐depleted yellow wine and ginger juice controls, orthogonal EVLPs purification and characterization, cargo‐depletion or reconstitution experiments, in vivo efficacy validation, and standardized quality‐control parameters for food‐grade GE‐EVLPs development.

## 5 Conclusion

In this study, all GE‐derived EVLPs obtained from different processing products exhibited excellent hemocompatibility and biosafety, complying with the stringent requirements for food‐grade nanomaterials. These results provide the first confirmation of the presence of EVLPs in processed GE products and clarify their potential for development as functional food ingredients. This study systematically compared the physicochemical properties of three types of EVLPs, including zeta potential, protein content, and active small molecule composition. Through a comprehensive evaluation incorporating cytotoxicity assays, hemolysis tests, and histopathological analysis of major organs, the biosafety of DGE‐EVLPs, WGE‐EVLPs, and GGE‐EVLPs was confirmed, thoroughly validating their safety as food‐grade nanomaterials. As a traditional processing technique, wine processing may contribute to nanoscale changes associated with improved cellular uptake and functional delivery potential of GE‐EVLPs, as supported by the BV2 uptake assay. This finding opens new avenues for developing processed GE‐based EVLPs as functional food ingredients to support neurological wellness. Future studies should focus on elucidating the key active constituents in WGE‐EVLPs and advancing their translational applications in the health food industry. This work aims to establish a scientific foundation for the food‐oriented development of processed GE and the resource utilization of edible plant‐derived EVLPs.

## Author Contributions

**Jia Yu:** investigation. **Honghong Jiao:** conceptualization. **Yuanyuan Qin:** writing – original draft, methodology, investigation. **Jingyu Weng:** project administration, conceptualization. **Hongbo Xu:** writing – review and editing, validation, funding acquisition, conceptualization. **Shuming Li:** writing – review and editing, project administration, investigation, funding acquisition, conceptualization. **Yuangui Yang:** funding acquisition, formal analysis. **Bo Li:** project administration, funding acquisition, conceptualization. **Xinyi Liu:** investigation. **Jiaofeng Wu:** conceptualization. **Ke Wang:** conceptualization, writing – review and editing.

## Funding

This work was supported by the National Natural Science Foundation of China (82405030, 82404903), the National Key Research and Development Program of China (2022YFD1602004), the Young Talent Support Program of Shaanxi Science and Technology Association (20250323), the Key project of the Shaanxi Provincial Department of Education (24JR052), the Science and Technology Youth Stars Project of Shaanxi University of Chinese Medicine (2024‐KJXX‐06), the Doctoral Science Foundation of Shaanxi University of Chinese Medicine (306‐171020323004), the “Double Chain Integration” Young and Middle‐aged Scientific Research Innovation Team Project of Shaanxi Provincial Administration of Traditional Chinese Medicine (2022‐SLRH‐YQ‐005), and the Shaanxi Provincial Key Research and Development Program (2023‐ZDLSF‐54).

## Ethics Statement

All procedures involving rats were approved by the Ethics Committee of Shaanxi University of Chinese Medicine and the experimental animal ethics batch number is SUSMDL20250225002.

## Conflicts of Interest

The authors declare no conflicts of interest.

## Supporting information


**Figure S1:** Standard curve for protein concentration determination.
**Figure S2:** UPLC‐MS total ion chromatograms of three EVLPs under AMIDE mode.
**Figure S3:** UPLC‐MS total ion chromatograms of three EVLPs under RP mode. (a–f) Representative TIC profiles acquired in (a–c) positive electrospray ionization (ESI+) mode and (d–f) negative electrospray ionization (ESI−) mode. The intensity scale (y‐axis) represents the total ion current detected across the monitored mass range at each time point (x‐axis).
**Figure S4:** Metabolomics analysis. (a) KEGG pathway analysis and (b) compound distribution score for DGE‐EVLPs, WGE‐EVLPs, and GGE‐EVLPs.
**Figure S5:** The HPLC chromatograms of the standard versus the three EVLPs.
**Figure S6:** CC in GO pathway enrichment map of potential target genes.
**Figure S7:** MF in GO pathway enrichment map of potential target genes.
**Figure S8:** Schematic illustration of the molecular docking between Kaempferol and the target proteins.
**Figure S9:** Protein concentration of DGE‐EVLPs, WGE‐EVLPs, and GGE‐EVLPs.
**Figure S10:** Representative H&E staining of major organs (heart, kidney, spleen, lung, and liver). Scale bar of 100 μm.
**Figure S11:** Fluorescence imaging of dye‐only negative control by PBS without EVLPs incubated with PKH67.
**Table S1:** Primer sequence for qPCR analysis.
**Table S2:** The average particle size, zeta potential, particle concentration, and protein concentration ratio of DGE‐EVLPs, WGE‐EVLPs and GGE‐EVLPs.

## Data Availability

The data that support the findings of this study are available from the corresponding author upon reasonable request.
