# Supplementary material for: Comparative Analysis of Extracellular Vesicle‐Like Particles From Different Processing Gastrodia elata Bl.: Physicochemical Properties, Biosafety, and Neuroprotection Potential as a Functional Food Ingredient
Source: Food Sci Nutr. 2026 Jun 23;14(6):e72044. doi: 10.1002/fsn3.72044 (PMC13289689; doi:10.1002/fsn3.72044)
Supplement: Supplementary file 1 — Figure S1: Standard curve for protein concentration determination. Figure S2: UPLC‐MS total ion chromatograms of three EVLPs under AMIDE mode. Figure S3: UPLC‐MS total ion chromatograms of three EVLPs under RP mode. (a–f) Representative TIC profiles acquired in (a–c) positive electrospray ionization (ESI+) mode and (d–f) negative electrospray ionization (ESI−) mode. The intensity scale (y‐axis) represents the total ion current detected across the monitored mass range at each time point (x‐axis). Figure S4: Metabolomics analysis. (a) KEGG pathway analysis and (b) compound distribution score for DGE‐EVLPs, WGE‐EVLPs, and GGE‐EVLPs. Figure S5: The HPLC chromatograms of the standard versus the three EVLPs. Figure S6: CC in GO pathway enrichment map of potential target genes. Figure S7: MF in GO pathway enrichment map of potential target genes. Figure S8: Schematic illustration of the molecular docking between Kaempferol and the target proteins. Figure S9: Protein concentration of DGE‐EVLPs, WGE‐EVLPs, and GGE‐EVLPs. Figure S10: Representative H&E staining of major organs (heart, kidney, spleen, lung, and liver). Scale bar of 100 μm. Figure S11: Fluorescence imaging of dye‐only negative control by PBS without EVLPs incubated with PKH67. Table S1: Primer sequence for qPCR analysis. Table S2: The average particle size, zeta potential, particle concentration, and protein concentration ratio of DGE‐EVLPs, WGE‐EVLPs and GGE‐EVLPs. [file FSN3-14-e72044-s001.docx]

**Supplementary Material**

**Comparative Analysis of Extracellular Vesicle-Like Particles From Different Processing *Gastrodia elata* Bl.: Physicochemical Properties, Biosafety, and Neuroprotection Potential As a Functional Food Ingredient**

Yuanyuan Qin^1,†^, Shuming Li^1,†,*^, Jia Yu^1^, Jingyu Weng^1^, Bo Li^1^, Xinyi Liu^1^, Ke Wang2^b,^Yuangui Yang^1^, Honghong Jiao^1^, Jiaofeng Wu^1^, Hongbo Xu^1,*^

^1^ *Shaanxi University of Chinese Medicine, Shaanxi Collaborative Innovation Center of Chinese Medicinal Resources Industrialization, 712083, Shaanxi, Xianyang, PR China*

^2^ *School of Medical Engineering, Haojing College of Shaanxi University of Science & Technology, 712046,* *Shaanxi, Xianyang P.R. China*

**Supplementary Text**

**Stabilization** **Analysis**

The gastrointestinal stabilization of three GE-EVLPs was performed as follows: 1.34 μL of s**imulated gastric fluid (SGF,** Yuanye Bio-Technology, Shanghai, China**)** were added to 1 mL of three GE-EVLPs and incubated at 37°C for 30 min. Then, 80 μL of simulated intestinal fluid **(SIF,** Yuanye Bio-Technology, Shanghai, China**)** were added and incubated at 37°C for 30 min. The stability of three GE-EVLPs was measured by analyzing the change in surface particle size versus charge after each incubation using a nanoparticle size potentiostat by DLS.

**HPLC**

A mixed standard solution was prepared from gastrodin, *p*-hydroxybenzyl alcohol, *p*-hydroxybenzaldehyde, and parishins A, B, C, and E (each at 1.00 mg/mL in 50% methanol). **Chromatographic separation was carried out on a Shimpack GIST C18 column (250 mm × 4.6 mm, 5 µm) maintained at 30°C.** The mobile phase consisted of 0.1% phosphoric acid in water (A) and acetonitrile (B), with a flow rate of 1.0 mL/min. The gradient program was set as follows: 0–10 min, 3–10% B; 10–15 min, 10–12% B; 15–25 min, 12–15% B; 25–40 min, 15% B; 40–50 min, 15–20% B; 50–55 min, 20–40% B; 55–60 min, 40–95% B; 60–65 min, 95% B; 65–70 min, 95–3% B; 70–80 min, 3% B. Detection was performed at 220 nm)

**Supplementary Figures**

**
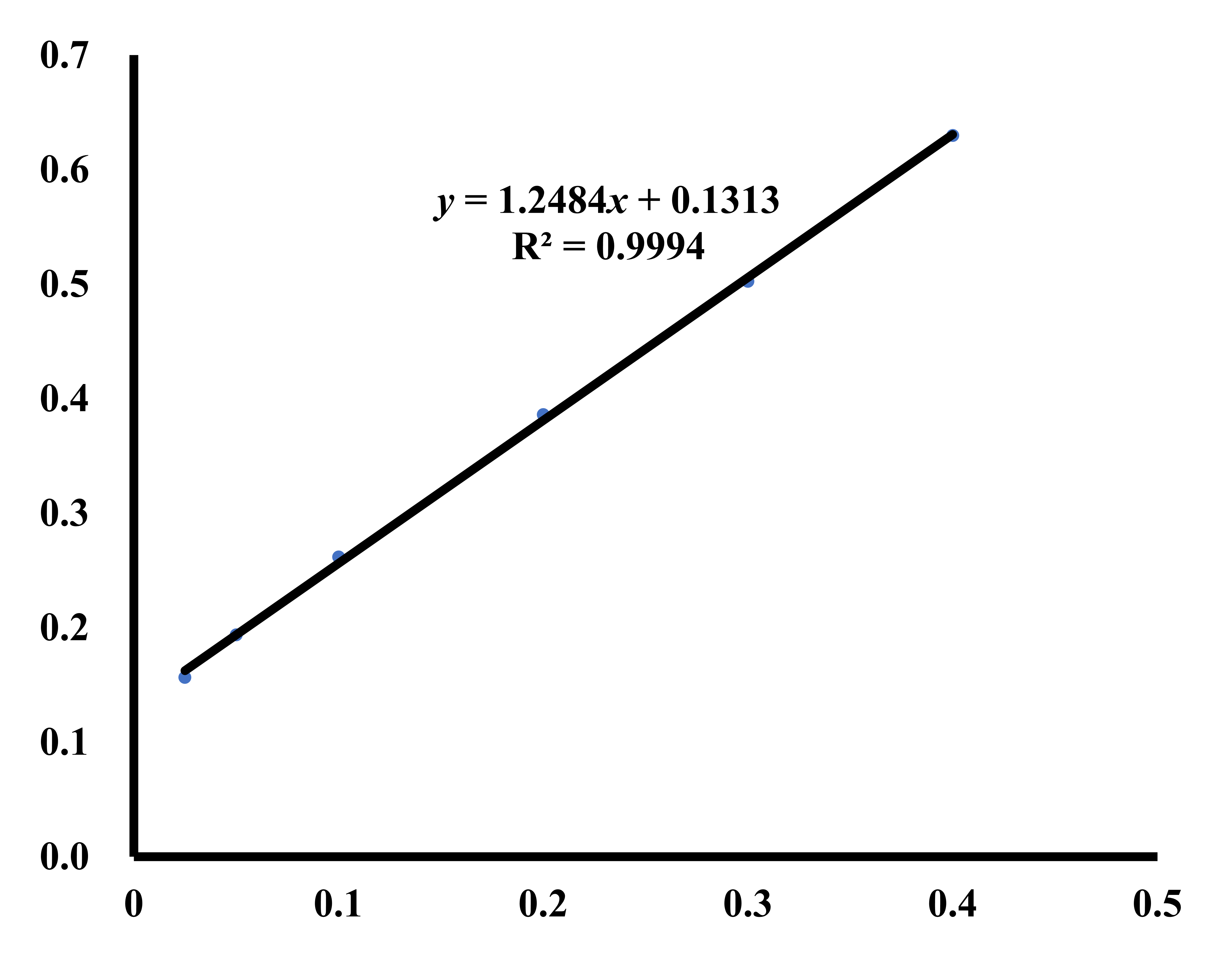
**

**FIGURE S1 Standard curve for protein concentration determination.**


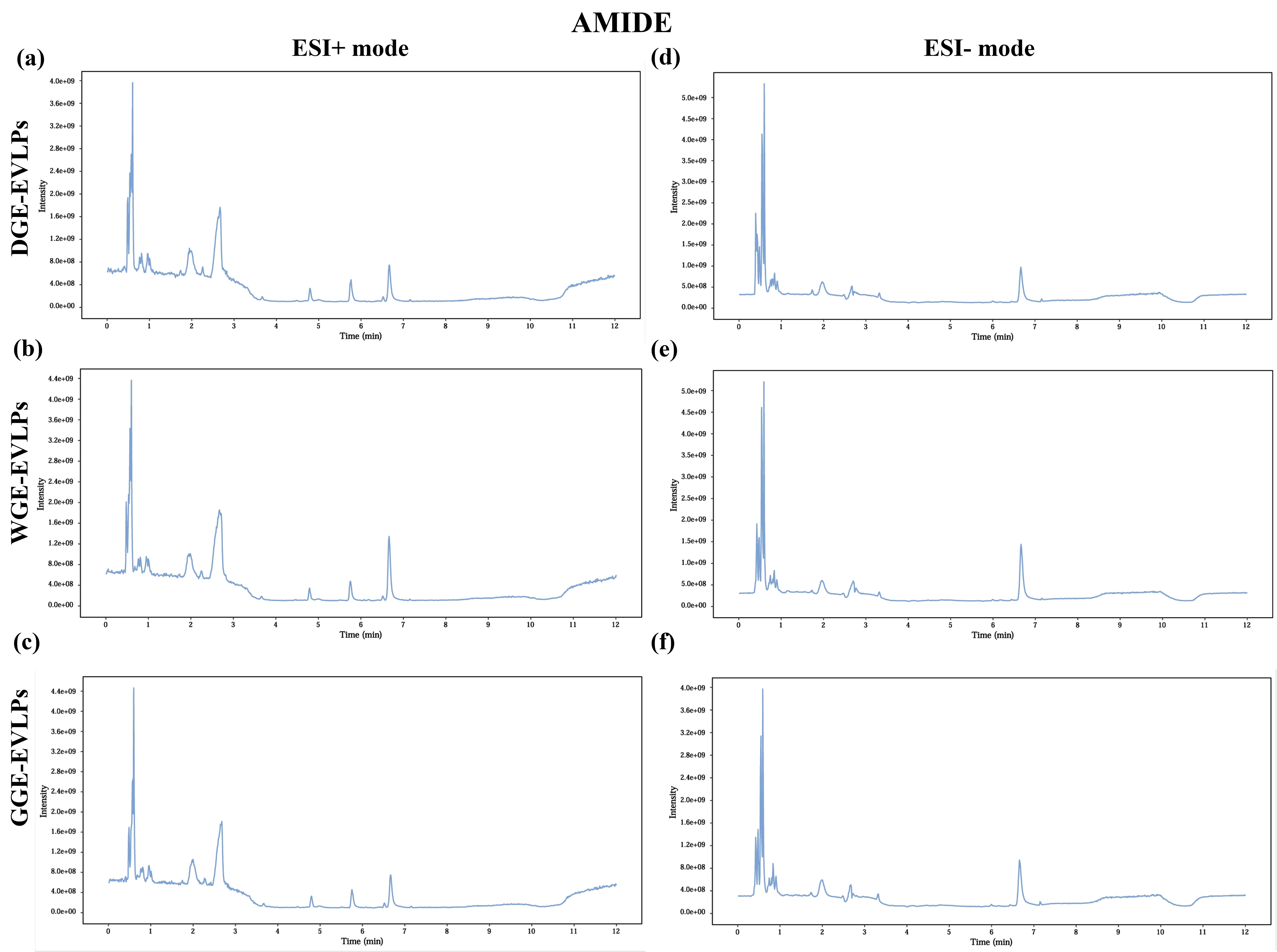


**FIGURE S2** UPLC-MS total ion chromatograms of three EVLPs under AMIDE mode.

(a–f) Representative TIC profiles acquired in (a-c) positive electrospray ionization (ESI+) mode and (d-f) negative electrospray ionization (ESI−) mode. The intensity scale (y-axis) represents the total ion current detected across the monitored mass range at each time point (x-axis).


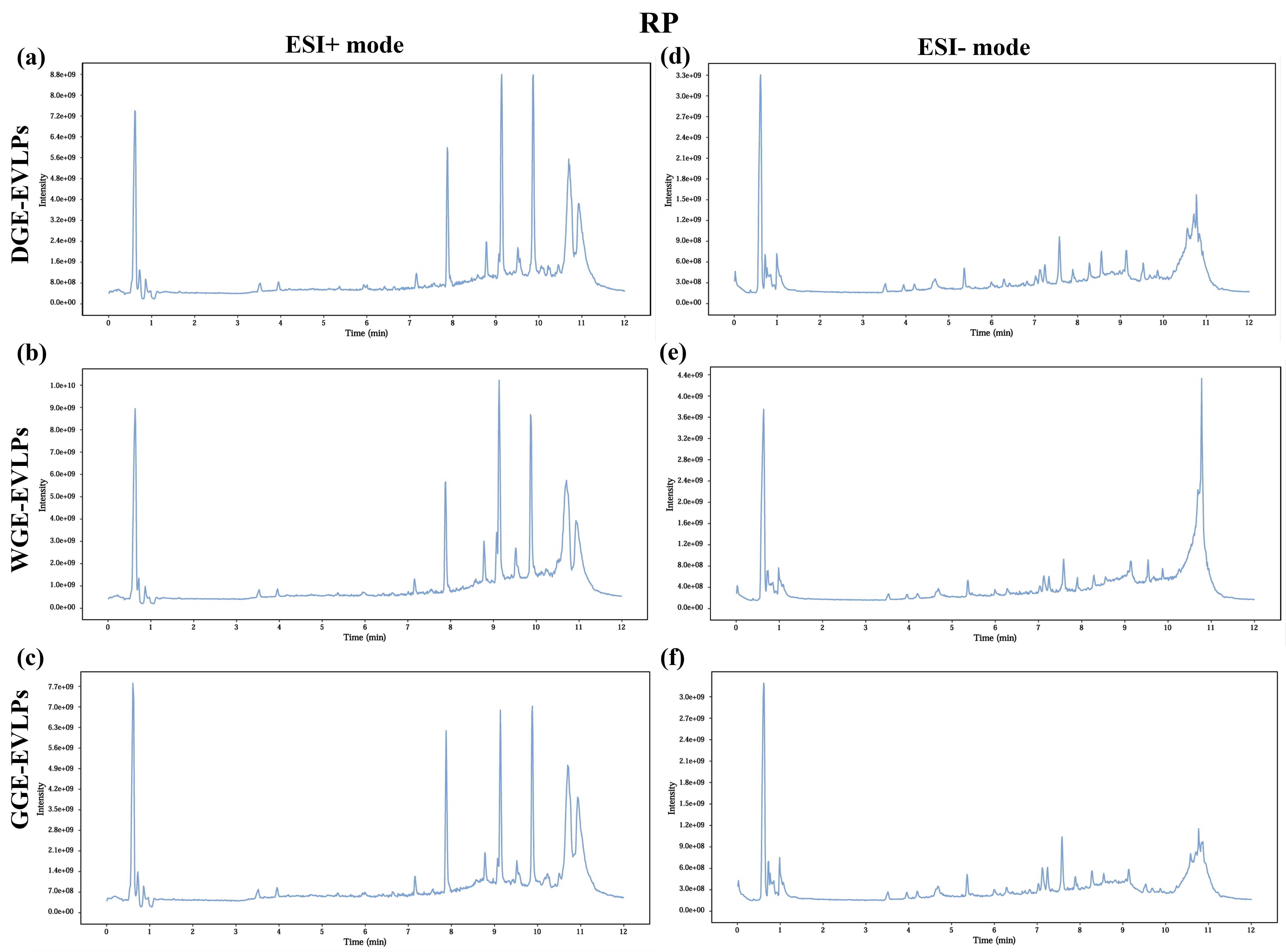


**FIGURE S3** UPLC-MS total ion chromatograms of three EVLPs under RP mode. (a–f) Representative TIC profiles acquired in (a-c) positive electrospray ionization (ESI+) mode and (d-f) negative electrospray ionization (ESI−) mode. The intensity scale (y-axis) represents the total ion current detected across the monitored mass range at each time point (x-axis).


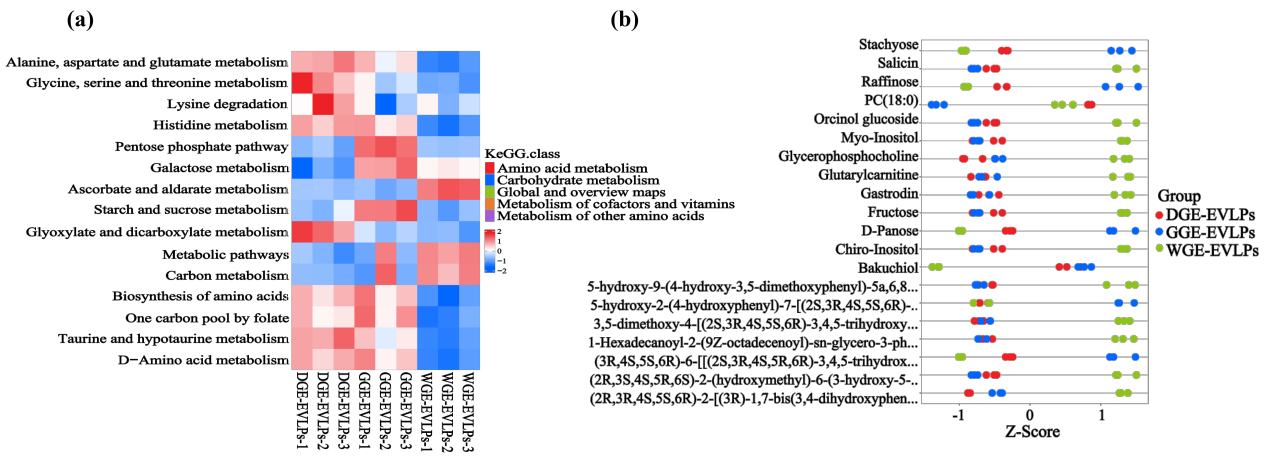


**FIGURE S4** Metabolomics Analysis. (a) **KEGG pathway analysis and (b) compound distribution score for DGE-EVLPs, WGE-EVLPs, and GGE-EVLPs.**


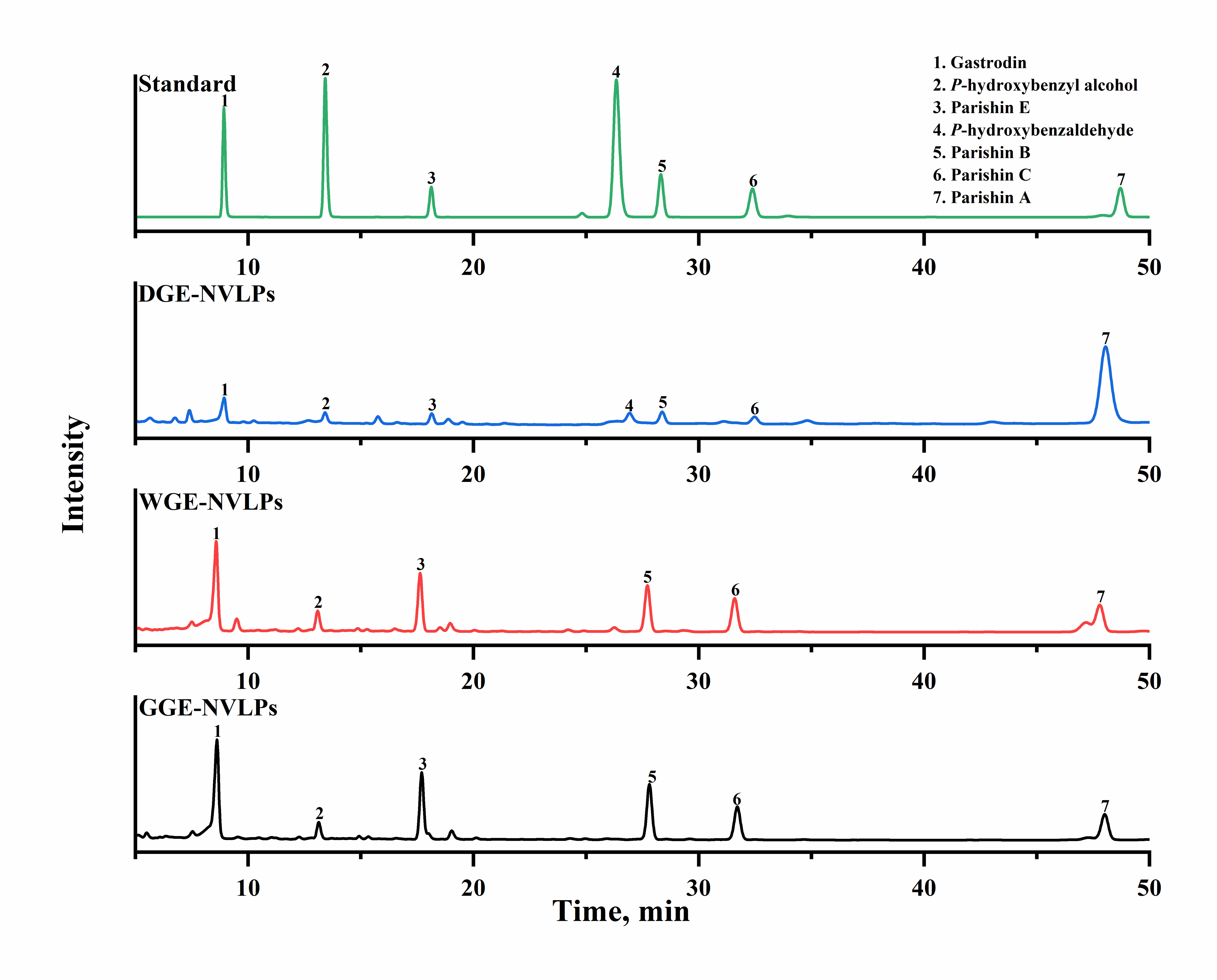
**FIGURE S5** The **HPLC chromatograms of the standard versus the three EVLPs.**


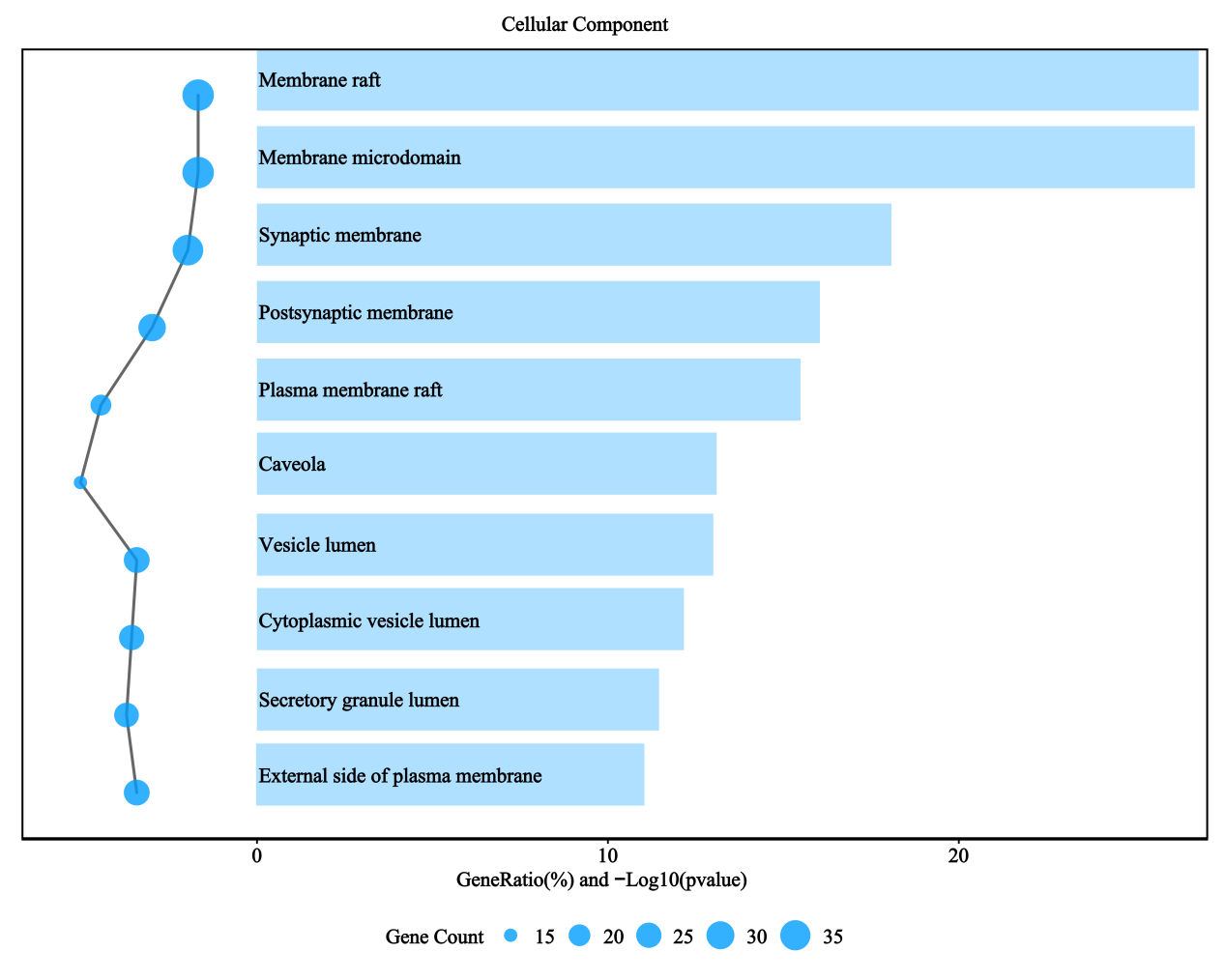
**FIGURE S6** CC in GO pathway enrichment map of potential target genes.

**
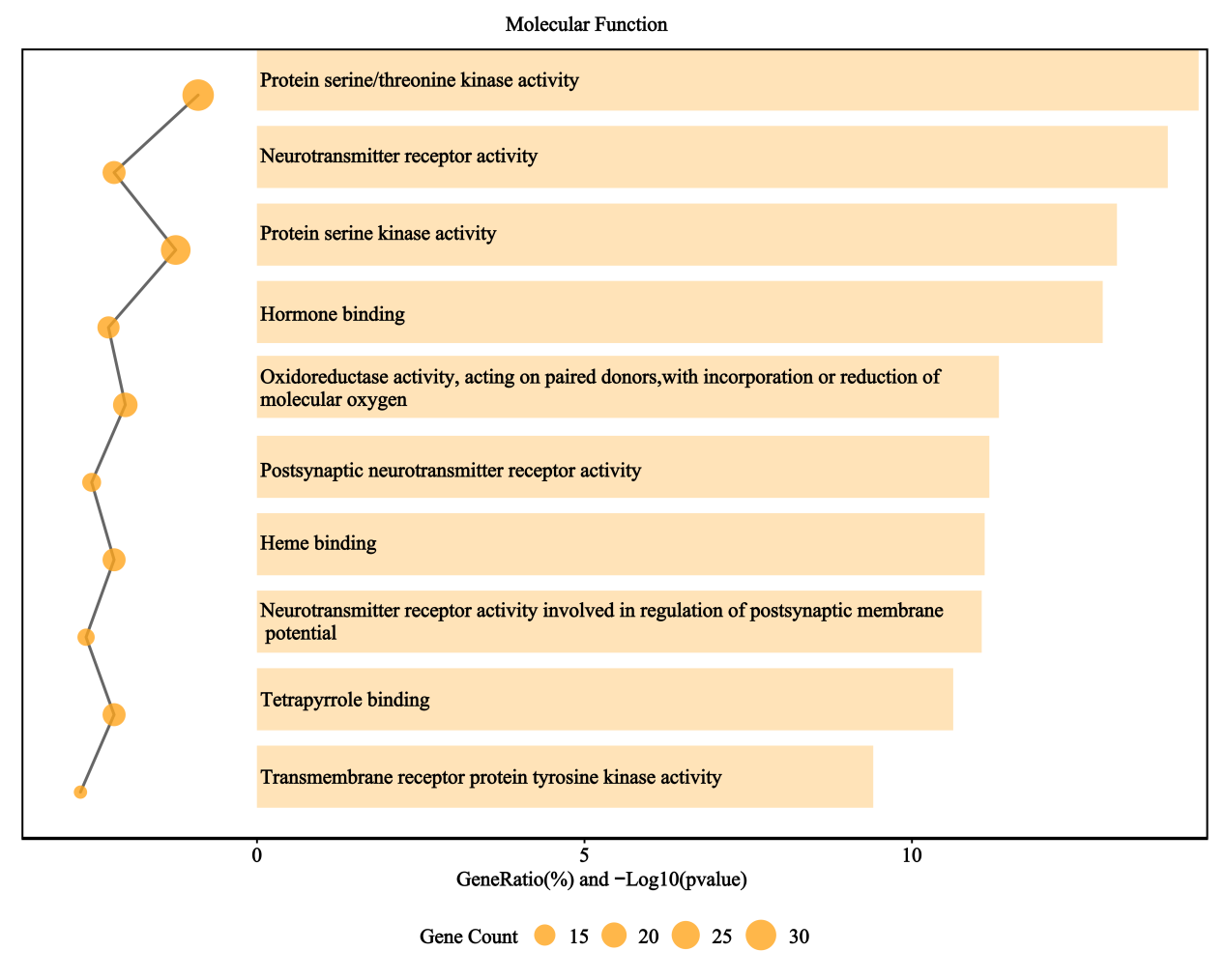
**

**FIGURE S7** MF in GO pathway enrichment map of potential target genes.

**
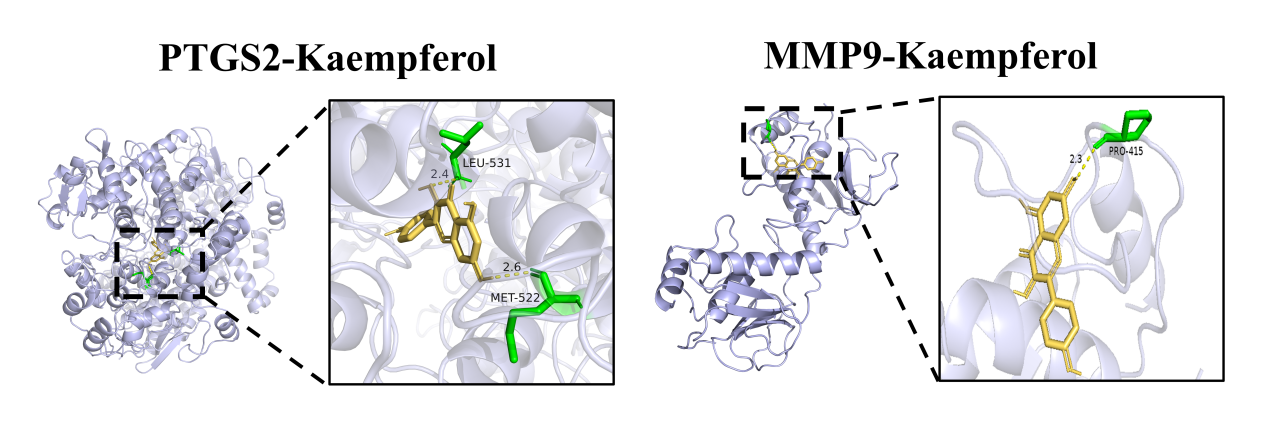
**

**FIGURE S8** Schematic illustration of the molecular docking between Kaempferol and the target proteins.


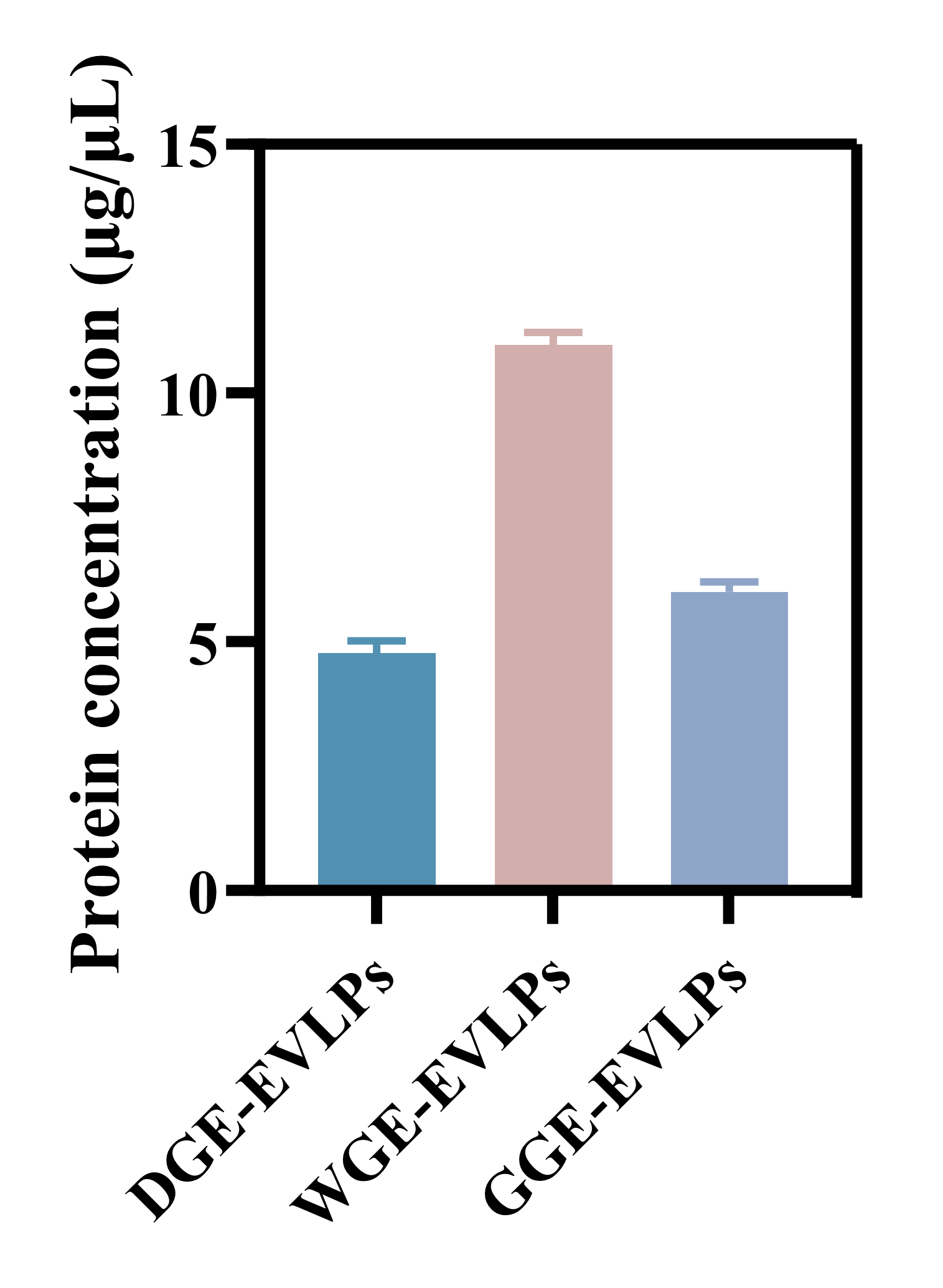


**FIGURE S9** **P**rotein **concentration of DGE-EVLPs, WGE-EVLPs, and GGE-EVLPs.**


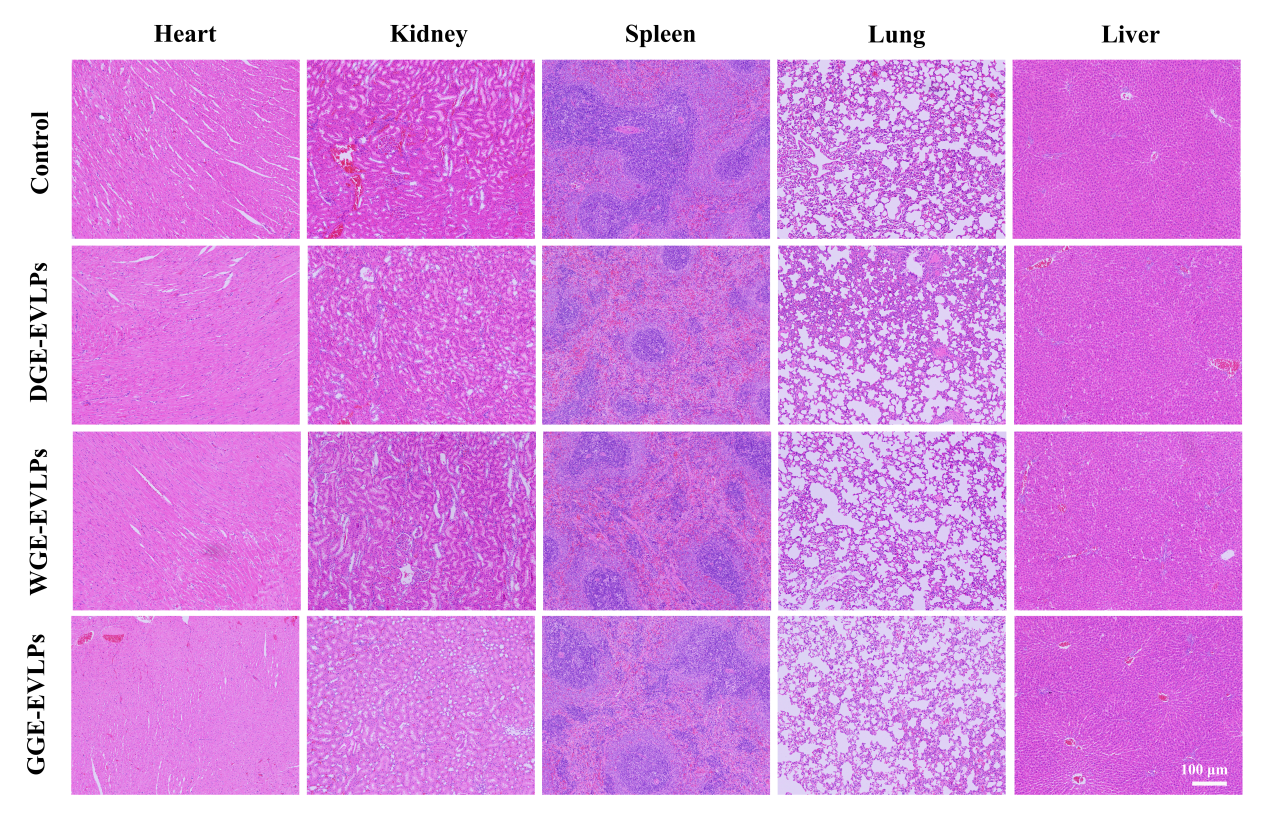


**FIGURE S10 Representative H&E staining of major organs (heart, kidney, spleen, lung, and liver).** Scale bar of 100 μm.

**FIGURE S11** Fluorescence imaging of dye-only negative control by PBS without EVLPs incubated with PKH67.

**TABLE S1.** Primer sequence for qPCR analysis

| **Gene** | **Direction** | **Sequence (5'-3')** |
| --- | --- | --- |
| **CD86** | Forward | ACGTATTGGAAGGAGATTACAGCT |
|  | Reverse | TCTGTCAGCGTTACTATCCCGC |
| **CD206** | Forward | GGGACTCTGGATTGGACTCA |
|  | Reverse | GCTCTTTCCAGGCTCTGATG |
| ***β*-actin** | Forward | CATTGCTGACAGGATGCAGAAGG |
|  | Reverse | TGCTGGAAGGTGGACAGTGAGG |
| **GAPDH** | Forward | GGGTGTGAACCACGAGAAAT |
|  | Reverse | CCTTCCACAATGCCAAAGTT |

**TABLE S2.** The average particle size, zeta potential, particle concentration, and protein concentration ratio of DGE-EVLPs, WGE-EVLPs and GGE-EVLPs.

|  | **Average particle size (nm)** | **Zeta potential**  **(mV)** | **Particle concentration**  **(particles/mL)** | **Protein concentration ratio (particles/μg)** |
| --- | --- | --- | --- | --- |
| **DGE-EVLPs** | 197.7 | -12.62±1.23 | 2.3×10^11^ | 4.8×10^9^ |
| **WGE-EVLPs** | 158.3 | -13.53±0.31 | 1.2×10^11^ | 1.1×10^9^ |
| **GGE-EVLPs** | 188.8 | -11.74±0.29 | 3.6×10^11^ | 5.8×10^9^ |
